# Diversity Survey of Macrofungal Resources in the Niyang River Basin Based on Soil High-Throughput Sequencing and Traditional Field Investigation

**DOI:** 10.3390/jof11120846

**Published:** 2025-11-28

**Authors:** Qianqian Yu, Congcong Liu, Ting Li, Shuaishuai Huang, Ruojin Liu, Yonghong Zhou

**Affiliations:** 1 Key Laboratory of Biodiversity and Environment on the Qinghai-Tibetan Plateau, Ministry of Education, Xizang University, Lhasa 850000, China; yq2301873395@163.com (Q.Y.); 18798907210@163.com (C.L.); s.huang1@utibet.edu.cn (S.H.); 2 School of Ecology and Environment, Xizang University, Lhasa 850000, China; 3 Xizang Museum of Natural Science, 10 Zangda East Road, Lhasa 850000, China; 4 State Key Laboratory of Applied Microbiology in Southern China, Guangdong Provincial Key Laboratory of Microbial Culture Collection and Application, Institute of Microbiology, Guangdong Academy of Sciences, Guangzhou 510070, China; liting@gdim.cn

**Keywords:** macrofungi, Niyang River Basin, high-throughput sequencing, fungal diversity, resource protection and development

## Abstract

This study conducted a comprehensive survey of macrofungal diversity in the Niyang River Basin by integrating soil high-throughput sequencing (HTS) with a traditional field investigation (TFI), aiming to evaluate the potential species pool and to assess the methodological complementarity of the two approaches. Compared with the TFI, the soil HTS method compensates for the impacts of climate and the growth phenology of macrofungi, and achieves a broader coverage in the investigation of macrofungal species. TFI is proven to be more effective in identifying conspicuous and parasitic fungi, whereas soil HTS is particularly advantageous in uncovering rare and previously unrecorded taxa. These findings highlight the necessity of methodological integration for accurately characterizing macrofungal diversity in complex ecosystems. The results not only establish a robust scientific foundation for the conservation and sustainable utilization of macrofungal resources on the Qinghai–Tibet Plateau, but also provide valuable methodological insights for future biodiversity assessments and conservation planning.

## 1. Introduction

It is estimated that there are approximately 2.2 to 3.8 million fungal species worldwide, while only about 120,000 species have been formally described, accounting for less than 8% of the total number of fungi [1]. Among them, macrofungi comprise roughly 450,000 species, with over 45,000 species reported from China, representing about 10% of the known global total [2]. Macrofungi are closely associated with human society. Many species are rich in proteins, bioactive polysaccharides, and other valuable compounds, and are widely used for both culinary and medicinal purposes. The conservation and utilization of genetic resources of macrofungi are of crucial importance. Their significance is manifested in multiple fields including ecology, medicine, agriculture, and industry, and they serve as key resources that support the development of relevant industries and ecological balance. With ongoing social and economic development, increasing attention has been paid to the economic and ecological value of macrofungi, leading to the establishment of stable edible and medicinal mushroom industries in many regions. For instance, *Lepista nuda* (wood blewit) has been reported to possess immunomodulatory, antibacterial, and antioxidant properties [3]; *Tremella fuciformis* (snow fungus) exhibits anticancer and antimicrobial activities [4]; and *Tricholoma matsutake* (matsutake mushroom) is rich in various bioactive compounds with antioxidant, antitumor, and immune-enhancing effects [5,6]. However, some macrofungi are highly toxic, capable of causing severe gastrointestinal and neurotoxic syndromes, such as *Amanita fulva*, *Chlorophyllum molybdites*, and *Inocybe fastigiata* [7]. Ecological diversity is an important component of biodiversity. It refers to the diversity of organisms in terms of ecology and habitat. No species can survive in isolation; all species can only grow in ecosystems composed of other species and their surrounding environment, thereby collectively forming the extremely complex ecological diversity on Earth. Macrofungi are a biological group of significant economic and ecological importance, and the study of their ecological types is of great significance for the conservation and scientific utilization of macrofungi [8].

Despite substantial research progress, the number of described fungal species still accounts for less than 8% of the estimated total, and the known diversity of macrofungi remains far below theoretical expectations. This highlights the significant knowledge gap in global macrofungal diversity, suggesting that a vast number of macrofungal resources in nature remain unexplored and undiscovered [9].

As one of the global biodiversity hotspots, Xizang harbors exceptionally rich macrofungal diversity due to its unique geographic and climatic conditions, which have fostered numerous endemic species and formed the highest-altitude genetic reservoir of macrofungal resources in the world. The Niyang River Basin, an important tributary of the Yarlung Zangbo River, features diverse vegetation types and complex ecosystems, providing favorable habitats for macrofungal growth [10]. However, the species composition and distribution of macrofungi in this basin remain insufficiently documented, with only a few systematic investigations reported to date [11]. To gain a deeper understanding of the macrofungal diversity in the Niyang River Basin, the present study integrated HTS with TFI to conduct the first comprehensive investigation and analysis of macrofungal communities across the basin.

In this study, we combined TFI with HTS to comprehensively reveal the macrofungal diversity of the Niyang River Basin by leveraging the complementary strengths of the two approaches. Furthermore, we compared the differences and similarities between these methods in terms of species diversity, aiming to provide a solid scientific foundation for the conservation and sustainable utilization of macrofungal germplasm resources. Ultimately, the findings of this study offer valuable data support and theoretical guidance for the conservation and utilization of macrofungal resources not only in the Qinghai–Tibet Plateau, but also in other ecologically similar regions.

## 2. Materials and Methods

### 2.1. Climatic Background of the Study Area

**Figure 1:**
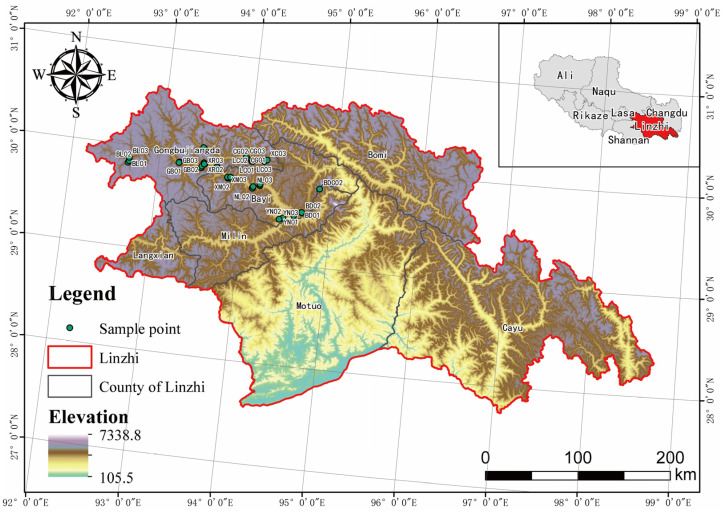
Map of sampling points in the Niyang River Basin; A total of 45 sampling sites were set up along the Niyang River Basin in this study.

The study area, the Niyang River Basin, is located within the ecological security barrier zone of southeastern Xizang, spanning 92°10′–94°35′ E and 29°28′–30°31′ N, with an elevation ranging from 2935 to 5030 m. It is the second-largest tributary of the Yarlung Zangbo River, covering a total area of approximately 1.78 × 10^4^ km^2^ and extending for about 309.02 km through Gongbo’gyamda County, Nyingchi County, and adjacent regions [12]. Situated on the southeastern margin of the Qinghai–Tibet Plateau, the basin lies in a zone of intense tectonic activity and exhibits typical alpine canyon landforms characterized by high elevations, thin air, and strong solar radiation [13]. The regional climate is influenced by the warm and humid air currents from the Indian Ocean and the cold and dry westerlies, resulting in a unique transitional mountain climate ranging from humid to semi-humid conditions. The mean annual temperature is 8.2 ± 0.5 °C, and the mean annual precipitation varies vertically between 600 and 900 mm, showing a distinct asynchrony between heat and moisture availability [14]. The upper and middle reaches of the Niyang River Basin are dominated by temperate coniferous forests, forming a transitional zone between the humid to semi-humid forest regions of eastern Tibet and the semi-arid shrublands of western Xizang. The lower reaches, located within the humid montane coniferous forest zone of the middle and lower Yarlung Zangbo River, are characterized by diverse vegetation types [15]. Vegetation gradually transitions from meadows and grasslands in the upper reaches to evergreen coniferous and deciduous broad-leaved forests downstream. Given the basin’s complex topography dominated by steep mountains and deep valleys, a random reconnaissance survey was conducted to assess terrain conditions, after which 45 representative sites were selected for plot-based field investigations (Figure 1 and Table S1).

### 2.2. Sample Collection

From July 2023 to October 2024, a macrofungal diversity survey was conducted across multiple locations in the Niyang River Basin, including Bailang Village, Gongbo’gyamda County, Xiri Village, Linze Village, Bangre Village, Qigong Village, Cuogao Township, Luochi Village, Xinchuo, Baiding Village, Ximixiga Mountain, Nianglu Village, Kadinggou, Yani Wetland, and Bidong Village. Considering both the growth conditions of macrofungi and the accessibility of local areas, the random survey method was adopted to collect the fruiting bodies of macrofungi. A random reconnaissance survey method was employed to record macrofungal fruiting bodies. The clean portions of each fruiting body were immediately preserved in liquid nitrogen for subsequent DNA extraction and amplification, while the remaining parts were labeled and stored in ventilated nylon mesh bags to prevent decay, followed by drying with a thermostatic dryer for long-term preservation as dry specimens. At each sampling site, soil samples were collected using the five-point sampling method. After removing surface litter and humus, soil was collected at a depth of 5–10 cm using a soil sampler. Three replicates were combined into a composite sample. The samples were sieved through a 2 mm mesh, placed in sterile centrifuge tubes, and the geographical coordinates, altitude, and vegetation type of each site were recorded. The macrofungal fruiting body samples preserved in liquid nitrogen were transported to the laboratory for DNA amplification and extraction. The soil samples were stored at −20 °C in portable outdoor freezers immediately after collection for short-term preservation, and upon arrival at the laboratory, they were subdivided and stored at −80 °C for long-term preservation and subsequent HTS analyses. All samples were transported on dry ice to the Illumina MiSeq PE300 platform (Shanghai Majorbio Bio-Pharm Technology Co., Ltd., Shanghai, China) for HTS.

### 2.3. Species Identification and Community Composition Analysis

In this study, the primer pairs designed for sequencing the macrofungi collected from the Niyang River Basin were ITS1F and ITS4R (5′-CTTGGTCATTTAGAGGAAGTAA-3′- and 5′-TCCTCCGCTTATTGATATGC-3′), targeting the ITS1F–ITS4R region, and sequencing was performed on the Illumina MiSeq platform (Shanghai Majorbio). For soil samples, the primers ITS1F and ITS2R (5′-CTTGGTCATTTAGAGGAAGTAA-3′- and 5′-GCTGCGTTCTTCATCGATGC-3′) were used to amplify the ITS1F–ITS2R region, also sequenced on the Illumina MiSeq platform (Shanghai Majorbio).

Using the DADA2 algorithm, firstly, the sequencing error model was learned to distinguish sequence variations caused by sequencing errors from the true sequence variations in microorganisms themselves; then, the paired-end reads were assembled, and the error sequences were corrected through statistical inference. Finally, each unique true sequence variant, Amplicon Sequence Variant (ASV), was output, and the ASVs aligned to chloroplast and mitochondrial sequences were removed.

The remaining ASVs were taxonomically classified by aligning them against the UNITE databases to identify macrofungi taxa. Subsequently, macrofungi species were screened and verified based on the World Fungal Classification System, GenBank, GBIF, Index Fungorum, and Flora Fungorum Sinicorum, among other references [16,17,18,19,20].

For the analysis of taxonomic composition, the numbers of families, genera, and species were counted using Microsoft Excel. Following the statistical approach of Tuliguer and Li Yu [21], families containing ≥10 macrofungi species were defined as dominant families, while genera containing ≥5 species were defined as dominant genera.

### 2.4. Floristic Analysis and Similarity Comparison

Based on the distribution information of each genus reported in the literature [22,23,24,25,26,27,28,29,30,31,32,33], the macrofungi in the Niyang River Basin were classified according to their floristic geographical components, thereby determining the floristic composition of macrofungi species in the basin.

For the similarity analysis of macrofungi in the Niyang River Basin, the following steps were taken. Firstly, the similarity between the macrofungi identified from soil samples and those obtained from field collections in the Niyang River Basin was evaluated. Secondly, the macrofungi of the Niyang River Basin were compared with those of the western Hubei region in the Yangtze River Basin [34], the Shengshan National Nature Reserve in the Heilongjiang River Basin [35], and the Wangtian’e Nature Reserve in the Yalu River Basin [36]. Finally, similarity analyses were conducted between the macrofungi of the Niyang River Basin and those of the Wumeng Mountains in Guizhou [37] and the Yuhe region in Gansu [38], which share comparable hydrothermal and climatic conditions. The calculation method followed that described by Wang Hesheng [39], using the formula S = ×100%, where S represents the similarity coefficient, a denotes the number of genera shared between two regions, and b and c represent the total number of genera in each respective region. A higher number of shared genera indicates greater similarity and a closer floristic relationship between regions, and vice versa [40,41]. Therefore, comparing the shared genera among different floras provides an effective means to elucidate the relationships among macrofungi communities across regions.

### 2.5. Ecological Types and Resource Assessment

According to the GBIF [18] and Index Fungorum databases, the ecological types of the 1056 macrofungal species involved in this study were classified (Table S5). Furthermore, following the approaches of Bao Haiying, Dai Yucheng, Tuliguer, Li Yu, and Wan Ning [42,43,44,45,46,47], this study classified economically valuable macrofungi species across the entire Niyang River Basin into four categories based on resource evaluation: edible mushrooms, edible-medicinal mushrooms, medicinal mushrooms, and poisonous mushrooms.

### 2.6. Threat Assessment of Macrofungi

Based on the assessment of threat levels for macrofungi provided in the Red List of China’s Biodiversity: Macrofungi Volume (2018) [48] jointly issued by the Ministry of Ecology and Environment of the People’s Republic of China and the Chinese Academy of Sciences [48]. The large fungal species found in the research institute were compared with the already assessed species in China Biodiversity Red List-Macrofungi Volume (2018) [48], so as to summarize the threat level of the large fungal species in this study.

## 3. Results

### 3.1. Macrofungal Species Composition

**Figure 2:**
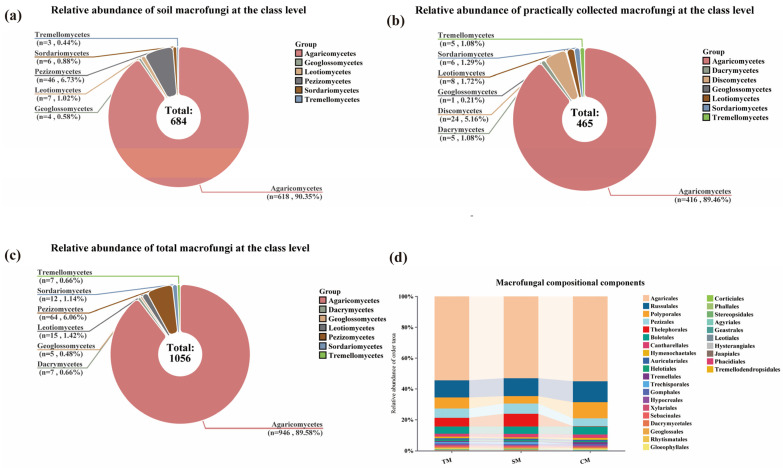
Macrofungal Species Composition in the Niyang River Basin. (**a**) Relative abundance of soil macrofungi at the class level, among them, the *Agaricomycetes* accounts for 90.35%; the *Pezizomycetes* accounts of 6.73%; the *Leotiomycetes* accounts of 1.02%, and the remaining taxa account for 1.9%. (**b**) Relative abundance of practically collected macrofungi at the class level, among them, the *Agaricomycetes* accounts for 89.46%; the *Discomycetes* accounts of 5.16%; the *Leotiomycetes* accounts of 1.72%, and the remaining taxa account for 3.66%. (**c**) Relative abundance of total macrofungi at the class level, among them, the *Agaricomycetes* accounts for 89.58%; the *Pezizomycetes* accounts of 6.06%; the *Leotiomycetes* accounts of 1.42%, and the remaining taxa account for 2.94%. (**d**) Macrofungal compositional components, at the *order* level, there are 24 orders of soil HTS macrofungi (SM), 20 orders of macrofungi actually collected (CM), and a total of 30 orders of macrofungi (TM).

Through HTS of soil samples, a total of 684 known macrofungi species were identified, belonging to 2 phyla, 6 classes, 24 orders, 89 families, and 200 genera. Among them, the *Ascomycota* comprised 4 classes, 6 orders, 17 families, 35 genera, and 63 species, accounting for 9.21% of the total macrofungi species in the soil. In contrast, the *Basidiomycota* included 2 classes, 18 orders, 72 families, 165 genera, and 621 species, representing 90.79% of the total (Figure 2a).

A total of 1538 macrofungi fruiting body samples were collected in the Niyang River Basin using the random reconnaissance survey method and were subjected to both molecular and morphological identification. Ultimately, 465 macrofungi species were identified, belonging to 2 phyla, 7 classes, 21 orders, 71 families, and 177 genera. Among them, the *Ascomycota* comprised 4 classes, 7 orders, 13 families, 26 genera, and 39 species, accounting for 8.39% of the total species collected, while the *Basidiomycota* included 3 classes, 14 orders, 58 families, 151 genera, and 426 species, representing 91.61% of the total (Figure 2b).

By combining HTS with TFI, a total of 1056 macrofungi species were identified in the Niyang River Basin, belonging to 2 phyla, 7 classes, 30 orders, 107 families, and 295 genera. Among them, the *Ascomycota* comprised 4 classes, 9 orders, 22 families, 51 genera, and 96 species, accounting for 9.09% of all macrofungi species, whereas the *Basidiomycota* included 3 classes, 21 orders, 85 families, 244 genera, and 960 species, representing 90.91% of the total (Figure 2c).

In terms of species richness, combining the results from both methods, a total of 1056 macrofungal species were confirmed in the Niyang River Basin, with 684 species identified via soil HTS and 465 species confirmed from TFI samples. These results indicate that the two approaches are complementary, providing valuable data for constructing a comprehensive macrofungal species inventory in the Niyang River Basin and offering a solid foundation for future ecological studies and the conservation of fungal diversity (Table S2).

### 3.2. Analysis of Dominant Families and Genera

#### 3.2.1. Dominant Families

**Figure 3:**
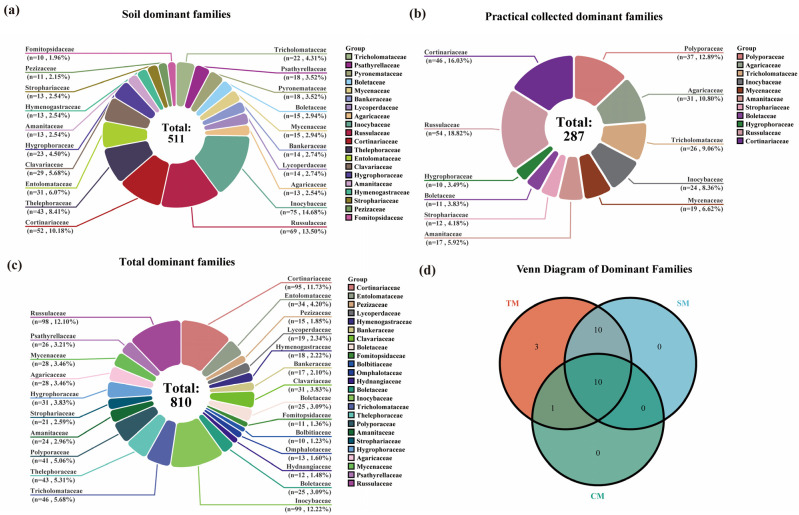
Dominant Families of Macrofungi in the Niyang River Basin. (**a**) Soil dominant families; Dominant families include: *Inocybeaceae*, *n* = 75, accounting for 14.68%-the highest proportion and the most dominant group in the soil; *Russulaceae*, *n* = 69, accounting for 13.50%; *Cortinariaceae*, *n* = 52, accounting for 10.18%; Other dominant families account for 61.64%. (**b**) Practical collected dominant families; Dominant families include: *Russulaceae*, *n* = 54, accounting for 18.82%, the most dominant group collected; *Cortinariaceae*, *n* = 46, accounting for 16.03%; *Polyporaceae*, *n* = 37, accounting for 12.89%; Other dominant families account for 52.26%. (**c**) Total dominant families. Dominant families include: *Inocybaceae*, *n* = 99, accounting for 12.22%; *Cortinariaceae*, *n* = 95, accounting for 11.73%; *Russulaceae*, *n* = 98, accounting for 12.10%;. Other dominant families account for 63.95%. (**d**) Venn Diagram of Dominant Families for Total Macrofungi (TM), Soil Macrofungi (SM) and Collected Macrofungi (CM).

Based on the analysis of soil HTS, the most species-rich dominant families of macrofungi in the Niyang River Basin were identified. The *Inocybaceae* was the richest, comprising 4 genera and 75 species, accounting for 10.96% of the total macrofungi species in the soil. This was followed by the *Russulaceae*, which included 4 genera and 69 species (10.09%), and the *Cortinariaceae*, comprising 7 genera and 52 species (7.60%). Overall, 20 dominant families were detected, encompassing 105 genera and 511 species. The dominant families accounted for 22.47% of all families present in the soil, and their included species represented 74.71% of the total macrofungi species identified in the soil Figure 3a).

In contrast, results based on TFI samples indicated that the *Russulaceae* was the largest dominant family, comprising 3 genera and 54 species, accounting for 11.61% of the total species collected. This was followed by the *Cortinariaceae*, which included 7 genera and 46 species (9.90%), and the *Polyporaceae*, comprising 14 genera and 37 species (7.96%). Overall, 11 dominant families with ≥10 species were identified among the collected samples, encompassing 71 genera and 287 species. These dominant families represented 15.49% of all families in the collected samples, and the species they included accounted for 61.72% of the total species collected (Figure 3b).

By integrating the results of soil HTS and TFI, the most species-rich dominant family of macrofungi in the Niyang River Basin was identified as the *Inocybaceae*, comprising 5 genera and 99 species, accounting for 9.38% of the total macrofungi species. This was followed by the *Russulaceae*, with 4 genera and 98 species (9.28%), and the *Cortinariaceae*, including 10 genera and 95 species (9.00%). Overall, there were 24 dominant families with ≥10 species, encompassing 166 genera and 810 species. These dominant families represented 22.43% of all families and 76.71% of the total macrofungi species in the Niyang River Basin (Figure 3c).

A comparison of the results obtained from the two methods indicates that the top three dominant families are largely consistent (Figure 3). However, 11 dominant families were identified from the TFI, whereas 21 dominant families were detected through soil HTS, indicating that the latter captured a greater variety of dominant families. Additionally, the *Polyporaceae*, which accounted for 37 species in the field surveys, was largely underrepresented in the soil sequencing results, with only 7 species detected (Table S3).

#### 3.2.2. Dominant Genera

**Figure 4:**
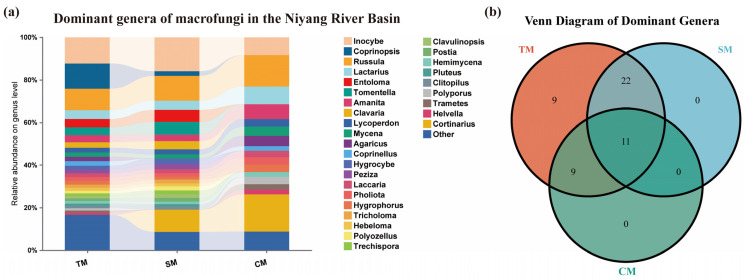
Dominant genera of macrofungi in the Niyang River Basin. (**a**) Certain genera exhibit extremely high proportions within specific groups (e.g., *Inocybe* accounts for over 9.80% in the SM group), indicating these genera represent the core dominant taxa within their respective groups and play a leading role in the fungal communities of that habitat/region. Multiple genera are distributed across the TM, SM, and CM groups but with significant variation in their proportions, reflecting the habitat specificity or widespread distribution of fungal genera. A bar chart with numerous thin color bands indicates high genus-level diversity within that group (coexistence of numerous low-abundance genera). Conversely, if a group is dominated by a few broad bands, it signifies low genus diversity and a concentration of dominant genera. (**b**) Venn Diagram of Dominant Genera for Total Macrofungi (TM), Soil Macrofungi (SM) and Collected Macrofungi (CM).

Based on the analysis of soil HTS, the most species-rich dominant genera of macrofungi in the Niyang River Basin were identified. The genus *Inocybe* was the richest, comprising 67 species, accounting for 9.80% of the total macrofungi species in the soil. This was followed by *Russula*, which included 49 species (7.16%), and *Cortinarius*, comprising 44 species (6.43%). Overall, 33 dominant genera were detected in the soil, encompassing 426 species. These dominant genera accounted for 16.50% of all families present in the soil and 62.28% of the total macrofungi species identified (Figure 4a).

In contrast, results from TFI samples indicated that the genus *Cortinarius* was the largest dominant genus, comprising 40 species, accounting for 8.60% of the total species collected. This was followed by *Russula*, which included 34 species (7.31%), and *Inocybe*, comprising 19 species (4.09%). Overall, 20 dominant genera with ≥5 species were identified among the collected samples, encompassing 229 species. These dominant genera represented 11.24% of all families in the collected samples, and the species they included accounted for 49.25% of the total species collected (Figure 4a).

By integrating the results of soil HTS and TFI, the most species-rich dominant genus of macrofungi in the Niyang River Basin was identified as *Inocybe*, comprising 83 species, accounting for 7.86% of the total macrofungi species. This was followed by *Cortinarius*, with 80 species (7.58%), and *Russula*, including 68 species (6.44%). Overall, there were 50 dominant genera with ≥5 species, encompassing 675 species. These dominant genera represented 16.95% of all genera and 63.92% of the total macrofungi species in the Niyang River Basin (Figure 4a).

By comparing the analysis results of the two methods, it can be seen that the top three dominant genera identified by both methods are basically consistent (Figure 4a). However, 20 dominant genera were identified from the TFI, whereas 33 dominant genera were detected through soil HTS, indicating that the latter captured a greater variety of dominant genera (Figure 4b).

### 3.3. Floristic Composition and Similarity Analysis of Macrofungi in the Niyang River Basin

#### 3.3.1. Analysis of Floristic Composition

**Figure 5:**
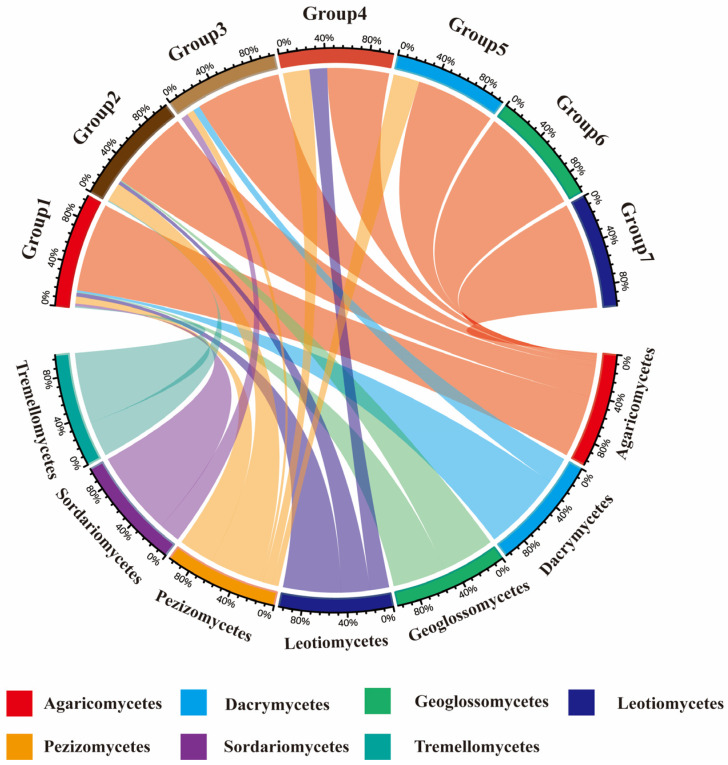
Geographical Flora of Macrofungi in the Niyang River Basin. The upper half represents geographical floristic groups, and the lower half represents taxonomic units of macrofungi. Group 1 to Group 7 represent, respectively: Cosmopolitan genera, North temperate genera, Pantropical genera, European-North American genera, Tropical-subtropical genera, Mediterranean-West Asia to Central Asia genera, China-endemic genera. *Agaricomycetes* exhibits extremely broad connectivity with multiple geographical faunal groups, particularly Group 1 and Group 4, indicating it is the most dominant and species-rich macrofungal group across all geographical faunas. *Pezizomycetes* and *Sordariomycetes* exhibit broad connecting bands within geographical groupings such as Group 4, representing key constituent groups within the region. Geographic Biogeography Groups 1 and 2: Primarily associated only with a few taxa such as the subclass *Agaricomycetes*, exhibiting relatively simple species composition and potentially demonstrating specific habitat selection effects.

The geographical distribution of fungi is generally classified based on the distribution patterns of genera or species. In this study, the geographical composition of macrofungi genera was analyzed. By integrating HTS and TFI, the 295 genera of macrofungi in the Niyang River Basin were roughly classified into seven types (Figure 5).

Cosmopolitan genera are those widely distributed across all continents without a specific center of distribution. In this study, 166 cosmopolitan genera were identified, accounting for 55.93% of the total macrofungi genera in the region, including notable genera such as *Agaricus*, *Amanita*, and *Antrodiella*. North-temperate genera are distributed in the temperate regions of the Northern Hemisphere, including Europe, North America, and temperate Asia. Ninety North-temperate genera were detected in this study, representing 30.51% of the total genera, with typical examples including *Aleuria*, *Amyloporia*, and *Arrhenia.* Pantropical genera are those distributed across tropical regions of both hemispheres, or extending into subtropical and temperate zones, but with distribution centers in the tropics. Fifteen pantropical genera were identified, accounting for 5.08% of the total genera, including *Caloboletus*, *Cotylidia*, and *Cruentomycena*. Europe-North America distributed genera refer to genera with a disjunct distribution in Europe and North America. In this study, 12 genera (4.07%) belonged to this category, including *Bondarzewia*, *Boubovia*, and *Hemimycena*. Tropical-subtropical genera are distributed in tropical regions of both hemispheres, sometimes extending to subtropical or temperate areas, but with distribution centers in the tropics. Eight genera (2.71%) fell into this category, including *Galiella*, *Ganoderma*, and *Geopyxis*. Mediterranean-West Asia to Central Asia genera are those distributed from the Mediterranean region through West or Southwest Asia to the Tibetan Plateau, Xinjiang, and Inner Mongolia in China. Only two genera were identified in this category: *Hypholoma* and *Macowanites*. Finally, two endemic genera to China were identified: *Spodocybe* and *Zangia*.

**Figure 6:**
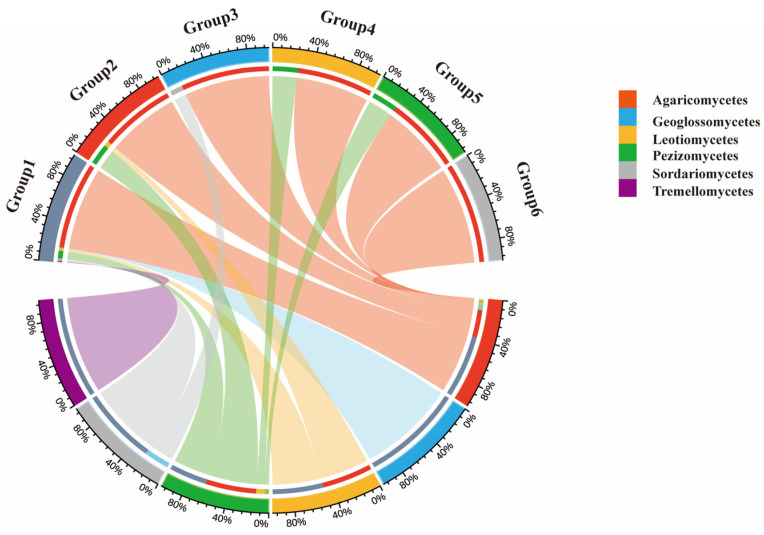
Geographical Distribution of Macrofungi in the Niyang River Basin. Group 1 to Group 6 represent, respectively: Cosmopolitan genera; North temperate genera; Pantropical genera; European-North American genera; Tropical-subtropical genera; Mediterranean-West Asia to Central Asia genera; *Agaricomycetes* exhibit extremely broad connectivity with all geographical faunal groups (Group 1–Group 6) and constitute a prominent proportion within each group. They represent the most dominant and widely distributed macrofungal group across all geographical faunal regions.

Analysis of the geographical composition of genera indicated that macrofungi in the Niyang River Basin are primarily composed of cosmopolitan genera (55.93%), followed by North-temperate genera (30.51%), pantropical genera (5.08%), and Europe-North America distributed genera (4.07%). Based on soil HTS, the soil-derived macrofungi were classified into six geographical types: cosmopolitan, North-temperate, pantropical, Europe-North America distributed, tropical-subtropical, and Mediterranean-West Asia to Central Asia (Figure 6). In contrast, the TFI classified the collected macrofungi into seven types, including all the six types identified by soil sequencing plus China-endemic genera, indicating a broader geographical coverage in the field survey. A comparison of the two methods shows that, in both approaches, the dominant component of the macrofungi flora is cosmopolitan, followed by North-temperate, pantropical, and Europe-North America distributed genera.

#### 3.3.2. Comparison of Similarity with Other Fungal Communities

The geographical composition of macrofungi in the Niyang River Basin is complex, with a rich taxonomic diversity. To further investigate the diversity of macrofungi in this region, the species identified in the Niyang River Basin were compared for similarity with previously reported macrofungi species from the E’xi region of the Yangtze River Basin, the Shengshan National Nature Reserve in the Heilongjiang River Basin, the Wangtian’e Nature Reserve in the Yalu River Basin, the Wumeng Mountains in Guizhou, and the Yu River in Gansu (Table 1).

The similarity analysis showed that the macrofungal communities identified in the Niyang River Basin by soil HTS and TFI shared 82 genera, with a similarity coefficient of 43.39%. Furthermore, when integrating the results of both methods, the macromycete flora of the Niyang River Basin exhibited varying degrees of similarity with other reported regional floras. The highest similarity was observed with the E’xi region of the Yangtze River Basin, sharing 159 genera and a similarity coefficient of 53.90%. This was followed by the Wumeng Mountains in Guizhou, with 116 shared genera and a similarity of 49.57%. In contrast, lower similarities were observed with the Yu River in Gansu (39.82%, 87 shared genera), the Wangtian’e Nature Reserve in the Yalu River Basin (38.89%, 77 shared genera), and the Shengshan National Nature Reserve in the Heilongjiang River Basin (36.94%, 70 shared genera).

### 3.4. Ecological Types and Resource Evaluation of Macrofungi in the Niyang River Basin

#### 3.4.1. Analysis of Ecological Types of Macrofungi

By combining HTS with TFI methods, a total of 1056 macrofungal species were identified in this survey. According to their nutrient acquisition methods, these species are classified into four major categories: saprotrophic fungi, symbiotic fungi, parasitic fungi, and facultatively parasitic/saprotrophic fungi (Table 2).

There are approximately 549 species of saprotrophic fungi, accounting for 51.99% of the total number of macrofungal species in the region. Most of them grow saprotrophically on dead parts of plant tissues or organs, and are commonly found in litter layers, fallen logs, dead branches, and dead branches of living standing trees. Their mycelia have a strong ability to decompose and utilize cellulose, hemicellulose, or lignin, causing white or brown rot in xylem. Generally, they are relatively easy to culture using tissue isolation methods. Symbiotic fungi refer to a group of fungi that form long-term, stable, and mutually beneficial symbiotic relationships with other living organisms. Their core characteristic is the two-way exchange of nutrients or living conditions between the fungi and their hosts, which distinguishes them from parasitic fungi and saprotrophic fungi. There are about 489 species of symbiotic fungi, making up 46.31% of the total macrofungal species in the region. Parasitic fungi are a group of fungi that must rely on living hosts to obtain nutrients and energy. Their core feature is plundering nutrients from the cells or tissues of living hosts, which often causes damage to the hosts. There are roughly 11 species of parasitic fungi, accounting for 1.04% of the total macrofungal species in the region. Facultatively parasitic/saprotrophic fungi are a group of fungi with “flexible” nutritional modes. They can not only live a parasitic life on living hosts but also switch to a saprotrophic life after the hosts die. They are a transitional group between strictly obligate parasitic fungi and strictly obligate saprotrophic fungi. There are about 7 species of facultatively parasitic/saprotrophic fungi, accounting for 0.66% of the total macrofungal species in the region (Table S5).

#### 3.4.2. Evaluation and Analysis of Macrofungal Resources

**Figure 7:**
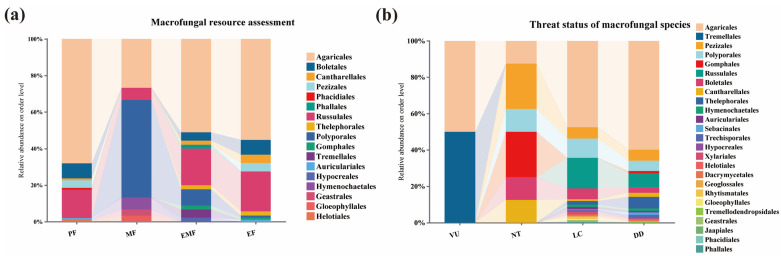
Economic Value and Threat Level of Macrofungi in the Niyang River Basin. (**a**) Macrofungal resource assessment. *Agaricales* account for a high proportion across all resource types, representing the “core group” of macrofungal resources. *Russulales* and *Boletales* are particularly prominent in EMF, reflecting their ecological symbiosis (forming mycorrhizae with plants) as a resource attribute. In PF, groups such as the *Russulales* also constitute a certain proportion, indicating that toxicity risks require attention during resource utilization. (**b**) Threat status of macrofungal species. *Agaricales* account for the highest proportion across all threat categories, indicating both their high species richness and broad coverage of threatened statuses. This may be related to their widespread distribution. Within the DD category, multiple groups constitute a significant proportion, reflecting information gaps in the conservation status of large fungal species. Enhanced surveys and research are required.

By combining HTS and TFI, a total of 260 economically valuable macrofungi species were identified in this survey, accounting for 24.62% of all macrofungi species recorded in the Niyang River Basin (Figure 7). Among these, 122 species were identified through traditional field surveys, while 138 species were detected by soil HTS. For reference regarding the potential macro-fungal resources in the Niyang River Basin.

A total of 97 toxic macrofungi species were identified in the Niyang River Basin, accounting for 9.19% of all macrofungi species. These species belonged to eight orders, including *Agaricales* 66 species, *Russulales* 15 species, *Boletales* 8 species, *Pezizales* 4 species, *Auriculariales* 1 species, *Cantharellales* 1 species, *Helotiales* 1 species, and *Phacidiales* 1 species.

A total of 30 medicinal macrofungi species were identified in the Niyang River Basin, accounting for 2.84% of all macrofungi species. These species belonged to six orders, including *Polyporales* 16 species, *Agaricales* 8 species, *Hymenochaetales* 2 species, *Russulales* 2 species, *Geastrales* 1 species, and *Gloeophyllales* 1 species.

A total of 45 species of edible and medicinal macrofungi were identified in the Niyang River Basin, accounting for 4.26% of all macrofungi species. These species belonged to 10 orders, including *Agaricales* 23 species, Russulales 9 species, Polyporales 4 species, *Boletales* 2 species, *Tremellales* 2 species, *Cantharellales* 1 species, *Gomphales* 1 species, *Hypocreales* 1 species, *Phallales* 1 species, and *Thelephorales* 1 species. Among these edible and medicinal species, some are toxic, including *Suillus viscidus* (L.) *Roussel*, *Pholiota lubrica* (Pers.) *Singer*, *Paxillus involutus* (Batsch) *Fr.*, *Mycena pura* (Pers.) *P.Kumm.*, *Gyroporus castaneus* (Bull.) *Quél.*, and *Bulgaria inquinans* (Pers.) *Fr.* [49]. Therefore, caution is advised when consuming these species.

A total of 87 edible macrofungi species were identified in the Niyang River Basin, accounting for 8.24% of all macrofungi species. These species belonged to nine orders, including *Agaricales* 48 species, *Russulales* 19 species, *Boletales* 7 species, *Cantharellales* 4 species, *Pezizales* 4 species, *Thelephorales* 2 species, *Auriculariales* 1 species, *Gomphales* 1 species, and *Polyporales* 1 species.

Among the 97 toxic macrofungi species identified in the Niyang River Basin, the potential poisoning types include gastrointestinal, neurotoxic, renal failure, hemolytic, hepatorenal damage, and respiratory-circulatory failure. Of these, 21 species have unclear toxicity types. Specifically, 28 species may cause gastrointestinal poisoning, 23 species neurotoxic effects, 6 species both gastrointestinal and neurotoxic effects, 5 species renal failure or hepatorenal damage, 2 species rhabdomyolysis, 1 species gastrointestinal and hemolytic poisoning, 1 species hemolytic poisoning, 1 species hepatorenal damage, respiratory-circulatory failure, and gastrointestinal poisoning, 1 species hepatorenal damage, gastrointestinal, neurotoxic, and hemolytic poisoning, and 1 species neurotoxic and respiratory-circulatory failure (Table S6).

The results showed that 122 species of economically valuable macrofungi were identified among the 465 species collected through TFI, accounting for 26.24% of the total species sampled. In contrast, 138 economically valuable species were identified among the 1056 species detected by soil HTS, representing 13.07% of the total soil-derived macrofungi species.

### 3.5. Threat Status of Macrofungal Species in the Niyang River Basin

By combining high-throughput sequencing with traditional field surveys, this study evaluated and summarized the threat status of 545 macrofungi species (Table S7), accounting for 51.61% of the total species identified. Among these, 2 species were classified as Vulnerable (VU), representing 0.19% of the total; 8 species as Near Threatened (NT), 0.56%; 303 species as Least Concern (LC), 28.69%; and 232 species as Data Deficient (DD), 21.97% (Figure 7). Additionally, 511 species have not yet been evaluated (Not Evaluated, NE) in the Red List and require further attention and assessment.

The two species classified as VU are *Naematelia aurantialba* (Bandoni & M. Zang) *Millanes* & *Wedin* and *Tricholoma matsutake* (S. Ito & S. Imai) *Singer.* The eight species classified as NT are *Gomphus orientalis R.H. Petersen* & *M. Zang*, *Ramaria distinctissima R.H. Petersen* & *M. Zang*, *Ramaria hemirubella Petersen* & *Zang*, *Ganoderma applanatum* (Pers.) *Pat.*, *Chroogomphus confusus Yan C. Li* & *Zhu L. Yang*, *Laccaria alba Zhu L. Yang* & *Lan Wang*, *Tuber alboumbilicum Y. Wang* & *Shu H. Li*, and *Tuber wenchuanense L. Fan* & *J. Z. Cao.* The 303 species categorized as LC are distributed across 22 orders, including *Agaricales* 144 species, *Russulales* 51 species, *Polyporales* 32 species, *Pezizales* 19 species, *Boletales* 18 species, *Thelephorales* 6 species, *Auriculariales* 4 species, *Hymenochaetales* 4 species, *Xylariales* 4 species, *Cantharellales* 3 species, *Rhytismatales* 3 species, *Dacrymycetales* 2 species, *Geoglossales* 2 species, *Gloeophyllales* 2 species, *Hypocreales* 2 species, *Geastrales* 1 species, *Helotiales* 1 species, *Jaapiales* 1 species, *Phacidiales* 1 species, *Phallales* 1 species, *Sebacinales* 1 species, and *Trechisporales* 1 species. A total of 232 species were classified as DD, belonging to 17 orders. These include *Agaricales* 139 species, *Russulales* 18 species, *Thelephorales* 15 species, *Pezizales* 14 species, *Polyporales* 13 species, *Boletales* 7 species, *Cantharellales* 5 species, *Gomphales* 3 species, *Hymenochaetales* 3 species, *Sebacinales* 3 species, *Trechisporales* 3 species, *Auriculariales* 2 species, *Helotiales* 2 species, *Hypocreales* 2 species, *Geoglossales* 1 species, *Tremellodendropsidales* 1 species, and *Dacrymycetales* 1 species.

The results showed that TFI identified a total of 465 macrofungi species, among which 188 species (40.43% of the total collected species) were evaluated in the China Biodiversity Red List—Macrofungi Volume (2018). Soil HTS 684 species, of which 357 species (52.19% of the total sequenced species) were evaluated in the Red List. Both TFI and soil HTS can provide assessments of the threat status of macrofungi in the studied sites, but each method has its advantages and limitations. In the Niyang River Basin, two species were classified as VU. Among them, *Naematelia aurantialba* (Bandoni & M. Zang) *Millanes & Wedin* was collected via TFI; it typically grows on tree trunks, either solitarily or in clusters, on decayed wood of broad-leaved trees in Fagaceae, Betulaceae, or mixed forests. Such species cannot be captured by soil sequencing. Other macrofungi that grow on tree trunks or decayed wood, such as *Ganoderma applanatum* (Pers.) *Pat.*, *Auricularia fuscosuccinea* (Mont.) *Henn.*, and *Dacrymyces australis Lloyd*, can only be sampled and surveyed through TFI. Data analysis further showed that soil HTS covered a broader range of macrofungi and was particularly effective for species categorized as DD, capturing 169 species, accounting for 72.84% of the total DD species.

## 4. Discussion

### 4.1. Comparison of Methods

TFI of macromycetes are often influenced by climatic conditions and incidental factors. To complement the preliminary specimen collection, soil samples were further collected from the study areas to explore potential macrofungal species that might have been missed during field surveys. HTS of these soil samples allowed for a more comprehensive assessment of the macromycete diversity in the Niyang River Basin. The results indicate that soil HTS and TFI each have distinct advantages and limitations in detecting macrofungal species. TFI provided more comprehensive coverage at the class level, identifying seven classes in total. Notably, the class *Dacrymycetes* was detected exclusively by TFI and included wood-inhabiting macrofungi such as the families *Auriculariaceae* and *Dacrymycetaceae*, which could not be captured through soil HTS. In contrast, HTS allowed for extensive coverage of soil fungal communities across a broader range of environments, revealing high species diversity within the soil. However, it was unable to detect tree-inhabiting fungi or non-sporulating fungi, which can only be surveyed and collected through TFI methods.

Comparison of data obtained from the two methods revealed that TFI and soil HTS shared 81 genera, resulting in a similarity coefficient of 42.86%. This indicates substantial differences in species composition and coverage between the two approaches. Specifically, TFI captured a greater diversity of species, particularly tree-inhabiting fungi, whereas soil HTS encompassed more macrofungal species growing in the soil, especially those well adapted to environmental conditions. Additionally, several genera that were dominant in the field-collected samples, including *Agaricus*, *Polyporus*, *Trametes*, *Helvella*, *Gymnopus*, *Pleurotus*, *Coprinellus*, *Clitocybe*, and *Leccinum*, were not detected among the dominant genera identified through soil sequencing. This discrepancy suggests that soil HTS identifies a broader range of dominant genera, whereas TFI captures more unique or specific taxa (Table S4). The combined approach provided a more comprehensive assessment of macrofungal diversity in the Niyang River Basin, effectively complementing the limitations inherent to each individual method.

TFI and HTS each have distinct advantages and limitations in the study of macrofungi diversity. TFIs rely on in situ collection of fungal specimens and species identification based on morphological characteristics. Their main advantage lies in providing detailed ecological context and direct species observations, which is particularly valuable for morphologically distinct fungi and for discovering rare or novel species [1]. However, this approach has inherent limitations: it is constrained by time and space, making it difficult to cover all areas and seasons; morphological identification requires high expertise and may overlook small or cryptic species [50]. Additionally, traditional methods have limited capacity to detect low-abundance or inconspicuous fungi, potentially underestimating species richness. In contrast, HTS analyzes environmental samples, such as soil or rhizosphere DNA, to reveal the composition of entire fungal communities, offering unique advantages for large-scale or complex ecosystems [51]. HTS can detect low-abundance fungi that are difficult to observe morphologically and can process large numbers of samples, providing comprehensive data on species distributions. Nevertheless, HTS also has limitations: it relies on extensive reference databases and bioinformatics tools, and insufficient database coverage can result in misidentification or undetected species [52]. Furthermore, HTS data analysis is complex and requires substantial computational resources and technical expertise. Overall, TFI and HTS are complementary in fungal diversity research. Integrating both approaches can overcome the limitations of each method and yield more comprehensive and accurate species data.

TFIs are influenced by various environmental factors, such as nutrient availability, temperature, moisture, air, and light, all of which directly affect the growth of macrofungi fruiting bodies [53]. Due to the temporal and spatial limitations of field sampling, HTS of soil, which captures DNA from spores and other residual materials, can effectively compensate for these shortcomings. This study not only provides a more systematic and comprehensive assessment of macrofungi diversity in the Niyang River Basin, complementing the results of TFI, but also demonstrates that integrating HTS with conventional field methods offers a novel approach for investigating fungal diversity.

### 4.2. Economic and Ecological Value

In the Niyang River Basin, 155 edible fungi, 55 fungi with both edible and medicinal value, 97 medicinal fungi, and 97 toxic fungi were identified. Given the relatively high diversity of edible, edible-medicinal, and medicinal fungi, local residents often consume these species in large quantities. However, many macrofungi exhibit similar morphological traits, increasing the risk of accidental ingestion of toxic species. Particular attention should be given to toxic fungi in the Niyang River Basin. For example, *Inocybe fibrosa* can cause hepatonephrotoxic, gastrointestinal, neurotoxic, and hemolytic poisoning, while *Naematelia subcorticalis* may induce hepatonephrotoxic, respiratory-circulatory failure, and gastrointestinal poisoning [7]. Among all toxic fungi, species causing gastrointestinal poisoning are the most prevalent 28 species, followed by those inducing neurotoxic effects, 23 species. Local authorities in surrounding cities and counties should strengthen public education on the food safety of wild edible fungi, advising residents to purchase mushrooms from regulated markets rather than collecting wild specimens themselves. In cases of accidental ingestion of toxic fungi, immediate emergency measures, such as induced vomiting, should be implemented, and medical attention should be sought promptly.

The ecological types of the 1056 macrofungal species in the Niyang River Basin are classified into 4 categories. Among them, saprotrophic fungi and symbiotic fungi total 1038 species, accounting for 98.3% of the total macrofungal species in this region. Additionally, when the macrofungi of the Niyang River Basin were assessed according to the Red List of China’s Biodiversity: Macrofungi Volume (2018) [48], 511 species had not yet been evaluated and therefore require subsequent assessment. Consequently, it is necessary not only to evaluate these unassessed macrofungi but also to implement systematic conservation and management measures for species classified as VU and NT. Both methods provide valuable data for assessing the economic value of macrofungi, and combining them allows for a more comprehensive representation of the economically important species at the study sites, offering more complete data support. In summary, while soil HTS provides broader coverage and is effective for identifying potential macromycetes in a site, it has limitations, particularly in detecting wood-decaying species. These species require TFI for sample collection and diversity assessment, whereas soil sequencing is more suitable for identifying and exploring potential macromycetes present in the soil.

### 4.3. Influence of Climate Conditions on Resource Richness and Uniqueness

The analysis of the floristic composition of macrofungi genera in the Niyang River Basin indicates that cosmopolitan and North Temperate genera are the dominant components, accounting for 86.78% of the total genera, while other floristic elements represent 13.22%. Overall, the Niyang River Basin exhibits a pronounced North Temperate distribution pattern. The region is located in the southeastern part of the Qinghai–Tibet Plateau and is characterized by a temperate, humid plateau climate with distinct mountainous climatic features. Recent studies have shown that, over time, extreme climatic conditions in the Niyang River Basin have generally exhibited a warm-wet trend. Both the amount, intensity, and frequency of extreme precipitation have increased, while the maximum and minimum extremes of temperature have risen, and the number of low-temperature days has decreased. Spatially, extreme climate changes in the basin show marked heterogeneity [54]. Climatic environment is the core driving factor for the diversity of macrofungi. The Qinghai–Tibet Plateau has a unique habitat, and extreme climatic conditions such as high altitude, strong radiation, low temperature, and large daily temperature difference have shaped the distinctive diversity of macrofungi, characterized by the enrichment of cold-tolerant species and the specific distribution of functional groups. Considering the factors affecting the growth of macrofungi fruiting bodies, these climatic changes in the Niyang River Basin are favorable for macrofungi growth and overall contribute to shaping the observed floristic distribution of macrofungi in the region.

A comparison of macrofungi species between the Niyang River Basin and other regions shows the highest similarity with the E’xi region of the Yangtze River Basin, indicating a high correspondence in dominant families and genera between the two areas. Considering local topography, geomorphology, and hydrothermal conditions, although the E’xi region and the Niyang River Basin are located in the central-eastern monsoon region and the Qinghai–Tibet Plateau, respectively, they share significant similarities in terrain, climate and vegetation, biodiversity, ecological function, and development patterns [55,56]. Consequently, the Niyang River Basin exhibits a relatively high proportion of shared genera with the E’xi region. The Niyang River Basin lies in a temperate, semi-humid monsoon climate zone, with hydrothermal and climatic conditions similar to those of the Wumeng Mountains in Guizhou and the Yuhe River in Gansu. However, the similarity indices between the Niyang River Basin and the Wumeng Mountains and Yuhe River are 49.57% and 39.82%, respectively, which may be attributed to the basin’s unique geographical location and the complexity of its climatic zone spectrum [53]. Across the five regions analyzed, similarity coefficients range from 36.94% to 53.90%, indicating relatively low similarity with most other regions. This pattern likely reflects the influence of vertical climatic zonation in the basin, resulting in diverse climate types and widespread species distributions, thereby highlighting the distinctiveness of macrofungi distribution patterns in the Niyang River Basin.

According to recent studies [57], the vegetation in the Niyang River Basin has generally shown a slight improvement in recent years, particularly along the riverbanks, where plant growth has markedly increased. This provides more favorable conditions for the growth and reproduction of macrofungi, contributing to the conservation of fungal species in the region and facilitating future specimen collection and inventory efforts. In this study, we conducted a macrofungi diversity survey in the Niyang River Basin by combining soil HTS with TFI, generating valuable data for the conservation, development, and utilization of these fungi. The results provide important insights into the diversity of macrofungi species in the basin and offer valuable data and methodological references for the sustainable protection and use of macrofungi resources on the Qinghai–Tibet Plateau. Moreover, integrating soil HTS with TFI enables the discovery of potential, previously unrecorded macrofungi species, offering a new approach and perspective for systematic exploration and study of undiscovered fungal species in this region.

## 5. Conclusions

In this study, we systematically investigated the macrofungi diversity of the Niyang River Basin by integrating soil HTS with TFI, revealing the region’s rich fungal resources. The combined results from both methods documented a total of 1056 macrofungi species, belonging to 2 phyla, 7 classes, 30 orders, 107 families, and 295 genera, highlighting the high species diversity in the area. Notably, 23 dominant families, including *Cortinariaceae Inocybaceae*, and *Russulaceae*, exhibited particularly prominent distributions across the basin.

The results indicate that climate change and improvements in vegetation within the Niyang River Basin have created more favorable conditions for the growth and reproduction of macrofungi, further promoting the conservation and sustainable utilization of fungal resources in the region. Despite limitations related to sampling time and locations, this study, by integrating data from soil HTS and TFI, effectively fills gaps inherent to conventional surveys and enriches knowledge of species diversity and distribution of macrofungi within the basin.

Furthermore, the study revealed that some macrofungi species in the Niyang River Basin are classified as VU or NT, highlighting the urgent need for targeted conservation and management measures. Future efforts should focus on the systematic protection, management, and sustainable utilization of macrofungi in this region, providing a more robust scientific basis for biodiversity conservation across the Qinghai–Tibet Plateau. 

## Figures and Tables

**Table 1:** Statistical Analysis of the Interrelationships Among Large Fungi and Regional Faunas in the Niyang River Basin.

Region	Phylum	Class	Order	Family	Genus	Species	Common Genus	Similarity Coefficient
Niyang River Basin	2	7	32	107	295	1056	295	100.00
Wumeng Mountains	2	8	20	75	173	469	116	49.57
Yuhe in Gansu	2	6	15	64	142	301	87	39.82
Heilongjiang River Basin	2	5	14	47	84	213	70	36.94
Yangtze River Basin	2	7	23	94	295	841	159	53.90
Yalu River Basin	2	7	17	48	101	161	77	38.89

**Table 2:** Statistics on the Number of Ecological Types of Macrofungi in the Niyang River Basin.

Ecological Type	Species	Percentage (%)
Saprotrophic	549	51.99
Symbiotic	489	46.31
Parasitic	11	1.04
Facultatively parasitic/saprotrophic	7	0.66

## Data Availability

The original contributions presented in this study are included in the article/Supplementary Materials. Further inquiries can be directed to the corresponding authors.

## Supplementary Materials

The following supporting information can be downloaded at: https://www.mdpi.com/article/10.3390/jof11120846/s1, Table S1. Niyang River Basin Sampling Site Information. Table S2. Statistics on the Number of Large Fungi Families, Genera, and Species in the Nyang River Basin. Table S3. Dominant Families of Macrofungi in the Nyang River Basin. Table S4. Statistics on Dominant Genera of Macrofungi in the Nyang River Basin. Table S5. Ecological Types of Macrofungi in the Niyang River Basin. Table S6. Toxic Fungal Species in the Nyang River Basin. Table S7. Statistics on the Threat Levels of Large Fungi in the Nyang River Basin.

## Abbreviations

The following abbreviations are used in this manuscript:

HTS	High-throughput Sequencing
TFI	Traditional Field Investigation
TM	Total Macrofungi
SM	Soil Macrofungi
CM	Collected Macrofungi
EF	Edible Fungi
MF	Medicinal Fungi
EMF	Edible and Medicinal Fungi
PF	Poisonous Fungi
VU	Vulnerable
NT	Near Threatened
LC	Least Concern
DD	Data Deficient
NE	Not Evaluated
