# Supplementary material for: Diversity Survey of Macrofungal Resources in the Niyang River Basin Based on Soil High-Throughput Sequencing and Traditional Field Investigation
Source: J Fungi (Basel). 2025 Nov 28;11(12):846. doi: 10.3390/jof11120846 (PMC12733894; doi:10.3390/jof11120846)
Supplement: Supplementary file 1 [file jof-11-00846-s001.zip › jof-3970431-supplementary.pdf]

Table S1. Niyang River Basin Sampling Site Information

Number	Longitude	Latitude	Elevation	Number	Longitude	Latitude	Elevation
BD01	94.665560°E	29.527462°N	3508.6m	LC03	94.057424°E	29.975067°N	3734.3m
BD02	94.672864°E	29.528391°N	3828.3m	CG01	93.986089°E	30.007311°N	3471.7m
BD03	94.586434°E	29.490475°N	4063.0m	CG02	93.986184°E	30.007270°N	3477.0m
YN01	94.431800°E	29.438238°N	3080.4m	CG03	93.976472°E	30.009457°N	3464.1m
YN02	94.465041°E	29.454028°N	2923.5m	QG01	93.496437°E	30.048911°N	3577.2m
YN03	94.427458°E	29.438452°N	3129.8m	QG02	93.497320°E	30.048262°N	3621.8m
KDG01	94.167424°E	29.747264°N	3053.4m	QG03	93.486634°E	30.056926°N	3710.5m
KDG02	94.170532°E	29.760312°N	3178.7m	BR01	93.660942°E	29.878373°N	3188.0m
KDG03	94.169861°E	29.762131°N	3240.0m	BR02	93.665002°E	29.883405°N	3488.6m
NL01	94.086927°E	29.716933°N	3121.3m	BR03	93.660460°E	29.881147°N	3697.4m
NL02	94.088880°E	29.716272°N	3219.1m	LZ01	93.489861°E	29.863907°N	3383.7m
NL03	94.095314°E	29.732221°N	3131.9m	LZ02	93.485928°E	29.857851°N	3471.3m
XM01	93.802244°E	29.793498°N	3133.2m	LZ03	93.491543°E	29.874142°N	3405.2m
XM02	93.797933°E	29.785375°N	3261.9m	XR01	93.521087°E	29.898324°N	3324.9m
XM03	93.795302°E	29.794417°N	3161.3m	XR02	93.511037°E	29.907496°N	3465.8m
BDC01	93.847281°E	29.778679°N	3173.2m	XR03	93.514764°E	29.896224°N	3389.8m
BDC02	94.848117°E	29.777254°N	3241.2m	GB01	93.227345°E	29.881431°N	3394.0m
BDC03	93.828322°E	29.797047°N	3126.5m	GB02	93.233324°E	29.888261°N	3388.7m
XC01	94.190896°E	29.990343°N	3835.3m	GB03	93.227653°E	29.881014°N	3439.3m
XC02	94.045025°E	29.980070°N	3718.2m	BL01	92.653817°E	29.823193°N	4236.0m
XC03	94.218923°E	30.008151°N	3830.1m	BL02	92.665313°E	29.839756°N	4116.6m
LC01	94.052386°E	29.975019°N	3693.4m	BL03	92.659247°E	29.871341°N	3972.1m
LC02	94.044131°E	29.982734°N	3715.3m				

Table S2. Statistics on the Number of Large Fungi Families, Genera, and Species in the Nyang River Basin

Family	Genus	Species	Family	Genus	Species
Inocybaceae	5	99	Entolomataceae	3	34
Russulaceae	4	98	Clavariaceae	5	31
Cortinariaceae	10	95	Hygrophoraceae	5	31
Tricholomataceae	21	46	Agaricaceae	9	28
Thelephoraceae	5	43	Mycenaceae	6	28
Polyporaceae	17	41	Psathyrellaceae	6	26

Boletaceae	15	25	Meripilaceae	3	3
Pyronemataceae	17	25	Rhizopogonaceae	1	3
Amanitaceae	2	24	Sclerodermataceae	1	3
Strophariaceae	8	21	Steccherinaceae	1	3
Lycoperdaceae	4	19	Clavicipitaceae	1	2
Hymenogastraceae	3	18	Cyphellaceae	2	2
Bankeraceae	4	17	Gautieriaceae	1	2
Pezizaceae	4	15	Gloeophyllaceae	1	2
Omphalotaceae	5	13	Gyroporaceae	1	2
Hydnangiaceae	1	12	Hyaloscyphaceae	1	2
Fomitopsidaceae	4	11	Hypoxylaceae	2	2
Bolbitiaceae	3	10	Leotiaceae	1	2
Helvellaceae	3	9	Omphidiaceae	1	2
Pleurotaceae	3	9	Paxillaceae	1	2
Cantharellaceae	3	8	Peniophoraceae	1	2
Hydnodontaceae	1	8	Phallaceae	1	2
Pluteaceae	1	8	Phanerochaetaceae	1	2
Meruliaceae	4	7	Stereopsidaceae	2	2
Auriscalpiaceae	2	6	Tremellaceae	1	2
Exidiaceae	2	6	Typhulaceae	2	2
Hydnaceae	1	6	Albatrellaceae	1	1
Hymenochaetaceae	4	6	Agyriaceae	1	1
Lyophyllaceae	3	6	Ascobolacea	1	1
Sarcoscyphaceae	4	6	Astraeaceae	1	1
Sebacinaceae	3	6	Bulgariaceae	1	1
Stereaceae	2	6	Ceratobasidiaceae	1	1
Helotiaceae	4	5	Cerrenaceae	1	1
Marasmiaceae	3	5	Coniophoraceae	1	1
Physalacriaceae	3	5	Crepidotaceae	1	1
Tuberaceae	1	5	Dermateaceae	1	1
Clavulinaceae	1	5	Discinaceae	1	1
Bondarzewiaceae	2	4	Eupolyporus	1	1
Dacrymycetaceae	4	4	Geastraceae	1	1
Ganodermataceae	2	4	Gomphidiaceae	1	1
Geoglossaceae	1	4	Hericiaceae	1	1
Gomphaceae	2	4	Hirschioporaceae	1	1
Ophiocordycipitaceae	1	4	Hyaloriaceae	1	1
Phaeotremellaceae	1	4	Hysterangiaceae	1	1
Schizoporaceae	3	4	Jaapiaceae	1	1
Suillaceae	1	4	Lentariaceae	1	1
Xylariaceae	1	4	Morchellaceae	1	1
Auriculariaceae	1	3	Podoscyphaceae	1	1
Corticiaceae	3	3	Sarcosomataceae	1	1
Cudoniaceae	2	3	Schizophyllaceae	1	1

Sclerotiniaceae	1	1	Tremellodendropsidaceae	1	1
Serpulaceae	1	1	Truncocolumellaceae	1	1
Sparassidaceae	1	1	Tubariaceae	1	1
Tapinellaceae	1	1	Total:	295	1056

Table S3. Dominant Families of Macrofungi in the Nyang River Basin

Dominant family	Species	Percentage(%)	Dominant family	Species	Percentage(%)
Inocybaceae	99	9.38	Pyronemataceae	25	2.37
Russulaceae	98	9.28	Amanitaceae	24	2.27
Cortinariaceae	95	9.00	Strophariaceae	21	2.00
Tricholomataceae	46	4.36	Lycoperdaceae	19	1.80
Thelephoraceae	43	4.07	Hymenogastraceae	18	1.70
Polyporaceae	41	3.88	Bankeraceae	17	1.61
Entolomataceae	34	3.22	Pezizaceae	15	1.42
Clavariaceae	31	2.93	Omphalotaceae	13	1.23
Hygrophoraceae	31	2.93	Hydnangiaceae	12	1.14
Agaricaceae	28	2.65	Fomitopsidaceae	11	1.04
Mycenaceae	28	2.65	Bolbitiaceae	10	0.95
Psathyrellaceae	26	2.46	Total:	810	76.71
Boletaceae	25	2.37			

Table S4. Statistics on Dominant Genera of Macrofungi in the Nyang River Basin

Dominant genera	Species	Percentage(%)	Dominant genera	Species	Percentage(%)
Inocybe	83	7.86	Hebeloma	10	0.95
Cortinarius	80	7.57	Tricholoma	10	0.95
Russula	68	6.44	Clavulinopsis	8	0.76
Lactarius	28	2.65	Hemimycena	8	0.76
Entoloma	26	2.46	Pluteus	8	0.76
Tomentella	25	2.37	Postia	8	0.76
Amanita	23	2.18	Polyozellus	8	0.76
Clavaria	17	1.61	Trechispora	8	0.76
Lycoperdon	15	1.42	Clitopilus	7	0.66
Agaricus	14	1.33	Helvella	7	0.66
Mycena	14	1.33	Polyporus	7	0.66
Hygrocybe	12	1.14	Trametes	7	0.66
Hygrophorus	12	1.14	Coprinellus	6	1.33
Laccaria	12	1.14	Clitocybe	6	0.57
Peziza	12	1.14	Gymnopilus	6	0.57
Pholiota	12	1.14	Gymnopus	6	0.57
Coprinopsis	10	0.95	Psathyrella	6	0.57

Sistotrema	6	0.57	Lepiota	5	0.47
Sarcodon	6	0.57	Mallocybe	5	0.47
Thelephora	6	0.57	Otidea	5	0.47
Clavulina	5	0.47	Panaeolus	5	0.47
Craterellus	5	0.47	Phellodon	5	0.47
Crepidotus	5	0.47	Pleurotus	5	0.47
Hydnellum	5	0.47	Stereum	5	0.47
Leccinum	5	0.47	Tuber	5	0.47
Lentinellus	5	0.47	Total：	677	64.11

Table S5. Ecological Types of Macrofungi in the Niyang River Basin

**Species**	**Trophic mode**
Agaricus abruptibulbus Peck, 1905	Saprotrophic
Agaricus arvensis Schaeff., 1774	Saprotrophic
Agaricus benesii (Pilát) Pilát	Saprotrophic
Agaricus bisporus (J.E. Lange) Imbach	Saprotrophic
Agaricus campestris L. :Fr.	Saprotrophic
Agaricus coccyginus M. Q. He & R. L. Zhao	Saprotrophic
Agaricus comtulus Fr., 1838	Saprotrophic
Agaricus deardorffensis Kerrigan 2016	Saprotrophic
Agaricus hingganensis R.L. Zhao & B. Cao 2020	Saprotrophic
Agaricus pilosporus Peck 1905	Saprotrophic
Agaricus subrutilescens (Kauffman) Hotson & D.E.Stuntz	Saprotrophic
Agaricus sylvaticus var. occidentalis (Kerrigan) Blanco-Dios,	Saprotrophic
Agaricus tibetensis J.L. Zhou & R.L. Zhao 2016,	Saprotrophic
Agaricus xanthodermus Genev.	Saprotrophic
Agrocybe pediades f. bispora	Saprotrophic
Agyrium aurantium W.Y. Zhuang & Zhu L. Yang	Saprotrophic
Aleuria aurantia (Pers.) Fuckel	Saprotrophic
Aleurodiscus wakefieldiae Boidin & Beller 1967	Saprotrophic
Amanita ahmadii Jabeen, I. Ahmad, M. Kiran, J. Khan & Khalid 2019,	Symbiotic
Amanita atrofusca Zhu L. Yang	Symbiotic
Amanita battarrae (Boud.) Bon	Symbiotic
Amanita breckonii Thiers & Ammirati	Symbiotic
Amanita brunneofuliginea Zhu L.Yang	Symbiotic
Amanita citrinoannulata Y.Y.Cui, Q.Cai & Zhu L.Yang	Symbiotic
Amanita citrinoindusiata Zhu L.Yang, Y.Y.Cui & Q.Cai	Symbiotic
Amanita crocea (Quél.) Singer	Symbiotic
Amanita flavipes S.Imai	Symbiotic
Amanita flavoconia G. F. Atk., 1902	Symbiotic

Amanita fritillaria(Berk.)Sacc.	Symbiotic
Amanita islandica Melot	Symbiotic
Amanita mangininana Har. & Pat	Symbiotic
Amanita muscaria (L.:Fr.) Pers.ex Hook.	Symbiotic
Amanita nivalis Grev.	Symbiotic
Amanita ochracea (Zhu L. Yang) Yang-Yang Cui, Qing Cai & Zhu L. Yang, 2018	Symbiotic
Amanita orientifulva Zhu L. Yang, M. Weiss & Oberw. 2004	Symbiotic
Amanita orsonii Ash.Kumar & T.N.Lakh.	Symbiotic
Amanita pakistanica Tulloss, S.H.Iqbal & Khalid	Symbiotic
Amanita subfrostiana Zhu L.Yang	Symbiotic
Amanita subglobosa Zhu L.Yang	Symbiotic
Amanita subjunquillea S.Imail	Symbiotic
Amanita vaginata (Bull.) Lam.	Symbiotic
Amyloporia sinuosa (Fr.) Rajchenb., Gorjón & Pildain	Saprotrophic
Antrodiella formosana T.T.Chang & W.N.Chou	Saprotrophic
Antrodiella zonata (Berk,)Ryvarden	Saprotrophic
Armillaria borealis Marxm. & Korhonen 1982,	Facultatively parasitic/saprotrophic
Armillaria cepistipes Velen., 1920	Facultatively parasitic/saprotrophic
Armillaria gallica Marxm. & Romagn., 1987	Facultatively parasitic/saprotrophic
Armillariella mellea (Vahl. Et Fr.)	Facultatively parasitic/saprotrophic
Arrhenia acerosa (Fr.) Kühner	Saprotrophic
Arrhenia epichysium (Pers.) Redhead, Lutzoni, Moncalvo & Vilgalys 2002	Saprotrophic
Arrhenia spathulata (Fr.) Redhead	Saprotrophic
Ascobolus carbonarius P. Karst.	Saprotrophic
Astraeus koreanus (V.J.Staněk) Kreisel	Symbiotic
Atheniella adonis (Bull.) Redhead, Moncalvo, Vilgalys, Desjardin & B.A.Perry	Saprotrophic
Atheniella delectabilis (Peck) Lüderitz & H.Lehmann	Saprotrophic
Aureoboletus quercus-spinosae Ming Zhang & T.H.Li	Symbiotic
Aureonarius callisteus (Fr.) Niskanen & Liimat. 2022	Symbiotic
Auricularia americana Parmasto & I.Parmasto ex Audet, Boulet & Sirard	Saprotrophic
Auricularia fuscosuccinea (Mont.) Henn., 1893	Saprotrophic
Auricularia villosula Malysheva 2014	Saprotrophic
Auriscalpium vulgare Gray	Saprotrophic
Bisporella citrina (Batsch) Korf & S.E. Carp. 1974,	Saprotrophic
Bisporella sulfurina(Qul.) S.E.Carp.	Saprotrophic
Bjerkandera adusta (Willd.,) P.Karst.	Saprotrophic
Bolbitius variicolor G.F. Atk., 1900	Saprotrophic
Boletus recapitulatus D.Chakr., K.Das, Baghela, S.K.Singh & Dentinger	Symbiotic
Boletus reticulatus Schaeff.	Symbiotic

Boletus reticuloceps (M.Zang, M.S.Yuan & M.Q.Gong) Q.B.Wang & Y.J.Yao	Symbiotic
Boletus sinoedulis B.Feng, Y.Y.Cui, J.P.Xu & Zhu L.Yang	Symbiotic
Bondarzewia mesenterica (Schaeff.) Kreisel	Saprotrophic
Bondarzewia tibetica B.K. Cui, J. Song & Jia J. Chen 2016,	Saprotrophic
Boubovia nicholsonii (Massee) Spooner & Y.J.Yao	Saprotrophic
Bovista aestivalis (Bonord.) Demoulin	Saprotrophic
Bovista plumbea Pers.	Saprotrophic
Bulgaria inquinans (Pers.) Fr.	Saprotrophic
Butyriboletus yicibus D.Arora & J.L.Frank	Symbiotic
Byssonectria fusispora (Berk.) Rogerson & Korf	Saprotrophic
Caloboletus panniformis (Taneyama & Har.Takah.) Vizzini	Symbiotic
Calocera tibetica F. Wu, L.F. Fan & Y.C. Dai 2021,	Saprotrophic
Calodon velutinum(Fr.) Quel.	Saprotrophic
Calonarius cupreorufus (Brandrud) Niskanen & Liimat.	Symbiotic
Calonarius pseudocupreorufus (Niskanen, Liimat. & Ammirati) Niskanen & Liimat.	Symbiotic
Calvatia gigantea (Batsch) Lloyd	Saprotrophic
Calyptella cernua (Schumach.) W.B.Cooke	Saprotrophic
Camarophyllopsis atrovelutina (Romagn.) Argaud	Saprotrophic
Campanella tristis (G. Stev.) Segedin 1993	Saprotrophic
Candolleomyces candolleanus (Fr.) D. Wächt. & A. Melzer 2020,	Saprotrophic
Cantharellus cibarius Fr.	Symbiotic
Ceraceomyces serpens (Tode) Ginns	Saprotrophic
Ceratellopsis acuminata (Fuckel) Corner	Saprotrophic
Cerrena unicolor (Bull.) Murrill	Saprotrophic
Chalciporus piperatus (Bull.) Bataille 1908	Symbiotic
Chamonixia caespitosa Rolland	Symbiotic
Cheilymenia theleboloides (Alb. & Schwein.) Boud.	Saprotrophic
Chroogomphus confusus Yan C.Li & Zhu L.Yang	Symbiotic
Cinereomyces lindbladii (Berk.) Jülich	Saprotrophic
Clavaria acuta Sowerby	Saprotrophic
Clavaria californica R.H.Petersen	Saprotrophic
Clavaria citrinorubra R.H.Petersen	Saprotrophic
Clavaria falcata Pers.	Saprotrophic
Clavaria flavipes Pers.	Saprotrophic
Clavaria flavostellifera Olariaga, Salcedo, P.P.Daniëls & Kautman	Saprotrophic
Clavaria fragilis Holmsk.	Saprotrophic
Clavaria fumosa Pers.	Saprotrophic
Clavaria globospora Kauffman	Saprotrophic
Clavaria greletii Boud.	Saprotrophic
Clavaria greletoides Arauzo & P.Iglesias	Saprotrophic

Clavaria pullei Donk	Saprotrophic
Clavaria redoleoalii R.H.Petersen	Saprotrophic
Clavaria sphagnicola Boud.	Saprotrophic
Clavaria tenuipes Berk. & Broome	Saprotrophic
Clavaria vermicularis Fr.	Saprotrophic
Clavaria zollingeri Lév.	Saprotrophic
Clavulicium delectabile (H.S.Jacks.) Hjortstam	Saprotrophic
Clavulina amethystina (Bull.) Donk	Symbiotic
Clavulina castaneipes (G.F.Atk.) Corner	Symbiotic
Clavulina coralloides (L.) J.Schröt.	Symbiotic
Clavulina reae Olariaga	Symbiotic
Clavulina rugosa (Bull.) J.Schröt.	Symbiotic
Clavulinopsis corniculata (Schaeff.) Corner	Saprotrophic
Clavulinopsis fusiformis (Sowerby) Corner	Saprotrophic
Clavulinopsis helvola (Pers.) Corner	Saprotrophic
Clavulinopsis hisingeri (P.Karst.) D.A.Reid	Saprotrophic
Clavulinopsis laeticolor (Berk. & M.A.Curtis) R.H.Petersen	Saprotrophic
Clavulinopsis luteoalba (Rea) Corner	Saprotrophic
Clavulinopsis luteonana Schild	Saprotrophic
Clavulinopsis trigonospora Franchi & M.Marchetti	Saprotrophic
Clitocybe bresadoliana Singer	Saprotrophic
Clitocybe diatreta (Fr.) P. Kumm., 1871	Saprotrophic
Clitocybe metachroa (Fr.) P.Kumm.	Saprotrophic
Clitocybe vibecina (Fr.) Quel. 1872	Saprotrophic
Clitopilus baronii Consiglio & Setti	Saprotrophic
Clitopilus fusiformis Di Wang & Xiao L. He 2017,	Saprotrophic
Clitopilus hobsonii (Berk.) P.D.Orton	Saprotrophic
Clitopilus passeckerianus (Pilát) Singer	Saprotrophic
Clitopilus prunulus (Scop.) P.Kumm.	Saprotrophic
Clitopilus scyphoides (Fr.) Singer	Saprotrophic
Clitopilus velutinus T.J.Baroni & Angelini	Saprotrophic
Collybia nuda (Bull.) Z.M. He & Zhu L. Yang 2023	Saprotrophic
Collybia odora (Bull.) Z.M. He & Zhu L. Yang, 2023 in [He Z et al. (2023)]	Saprotrophic
Collybia phyllophila (Pers.) Z.M. He & Zhu L. Yang, 2023 in [He Z et al. (2023)]	Saprotrophic
Collybiopsis biformis (Peck) R.H.Petersen	Saprotrophic
Collybiopsis subnuda (Ellis ex Peck) R.H. Petersen, 2022	Saprotrophic
Coniophora fusispora (Cooke & Ellis) Cooke	Symbiotic
Conocybe cylindracea Maire & Kühner ex Singer	Saprotrophic
Conocybe dumetorum (Velen.) Svrček	Saprotrophic
Conocybe pilosella (Pers.) Kühner	Saprotrophic
Conocybe utricystidiata (Enderle & H.-J. Hübner) Somhorst 2013	Saprotrophic

Cookeina insititia (Berk. & M.A.Curtis) Kuntze	Saprotrophic
Coprinellus disseminatus (Pers.) J.E.Lange	Saprotrophic
Coprinellus mcaceus (Bull.) Vilgalys et al.	Saprotrophic
Coprinellus micaceus (Bull.) Vilgalys, Hopple & Jacq.Johnson	Saprotrophic
Coprinellus ovatus M. Kamran & S. Jabeen 2020	Saprotrophic
Coprinellus radians (Desm.) Vilgalys, Hopple & Jacq.Johnson	Saprotrophic
Coprinellus xanthothrix (Romagn.) Vilgalys, Hopple & Jacq. Johnson 2001,	Saprotrophic
Coprinopsis atramentaria (Bull.) Redhead, Vilgalys & Moncalvo	Saprotrophic
Coprinopsis echinospora (Buller) Redhead, Vilgalys & Moncalvo	Saprotrophic
Coprinopsis filamentifera (Kühner) Redhead, Vilgalys & Moncalvo	Saprotrophic
Coprinopsis laanii (Kits van Wav.) Redhead, Vilgalys & Moncalvo	Saprotrophic
Coprinopsis lagopides (P.Karst.) Redhead, Vilgalys & Moncalvo	Saprotrophic
Coprinopsis lagopus (Fr.) Redhead, Vilgalys & Moncalvo	Saprotrophic
Coprinopsis nivea (Pers.) Redhead et.al	Saprotrophic
Coprinopsis scobicola (P.D.Orton) Redhead, Vilgalys & Moncalvo	Saprotrophic
Coprinopsis stercorea (Fr.) Redhead, Vilgalys & Moncalvo	Saprotrophic
Coprinopsis xenobia (P.D.Orton) Redhead, Vilgalys & Moncalvo	Saprotrophic
Coprinus comatus (O.F.Müll.) Pers.	Saprotrophic
Cordyceps cylindrica Petch	Parasitic
Cordyceps ophioglossoides (Ehrh.) Link	Parasitic
Coriolopsis trogii (Berk.) Domański 1974	Saprotrophic
Corticium boreoroseum Boidin & Lanq.	Saprotrophic
Cortinarius acutispissipes Rob. Henry 1981	Symbiotic
Cortinarius albocyaneus Fr., 1863	Symbiotic
Cortinarius alboviolaceus (Pers.) Fr.	Symbiotic
Cortinarius alpinus Boud.	Symbiotic
Cortinarius anomalus (Fr.) Fr.	Symbiotic
Cortinarius anthracinus (Fr.) E.Berger	Symbiotic
Cortinarius armillatus (Fr.) Fr.	Symbiotic
Cortinarius badioflavidus Ammirati, Beug, Niskanen, Liimat. & Bojantchev 2016,	Symbiotic
Cortinarius balaustinus Fr.	Symbiotic
Cortinarius balteatialutaceus Kytöv., Liimat. & Niskanen	Symbiotic
Cortinarius betuletorum M.M.Moser	Symbiotic
Cortinarius borealis Bidaud	Symbiotic

Cortinarius bridgei Ammirati, Niskanen, Liimat., Bojantchev & L. Fang 2017	Symbiotic
Cortinarius caesioarmeniacus Kytöv., Niskanen & Liimat. 2014	Symbiotic
Cortinarius campester Reumaux	Symbiotic
Cortinarius canabarba Moser	Symbiotic
Cortinarius caninus (Fr.) Fr.	Symbiotic
Cortinarius castaneopallidus Carteret	Symbiotic
Cortinarius castaneus (Bull.) Fr.	Symbiotic
Cortinarius centrirufus Kytöv., Niskanen & Liimat.	Symbiotic
Cortinarius cinnamomeus (L.) Fr.	Symbiotic
Cortinarius citrinoolivaceus Mos.	Symbiotic
Cortinarius claricolor (Fr.) Fr.	Symbiotic
Cortinarius croceus (Schaeff.) Gray	Symbiotic
Cortinarius desertorum (Velen.) G. Garnier 1991	Symbiotic
Cortinarius dolabratus Fr. 1838	Symbiotic
Cortinarius duracinus f. iners	Symbiotic
Cortinarius epipurrus Chevassut & Rob.Henry	Symbiotic
Cortinarius flabellus (Fr.) Fr.	Symbiotic
Cortinarius fuscescens Kytöv., Niskanen & Liimat.	Symbiotic
Cortinarius gentilis (Fr.) Fr.	Symbiotic
Cortinarius glaucopoides Kauffman	Symbiotic
Cortinarius glaucopus (Schaeff.) Fr.	Symbiotic
Cortinarius helobius Romagn.	Symbiotic
Cortinarius hinnuleoarmillatus Reumaux 1989	Symbiotic
Cortinarius hinnuleocervinus Niskanen, Liimat. & Ammirati 2017	Symbiotic
Cortinarius hirtus (Velen.) G.Garnier	Symbiotic
Cortinarius illuminus Fr.	Symbiotic
Cortinarius imbutus Fr.	Symbiotic
Cortinarius kranabetteri Niskanen, Liimat., Harrower, Ammirati & Dima	Symbiotic
Cortinarius longipes Peck	Symbiotic
Cortinarius mirandus Moënne-Locc. & Reumaux	Symbiotic
Cortinarius mucicola A.H.Sm.	Symbiotic
Cortinarius multiformis Fr.	Symbiotic
Cortinarius murinascens Kytöv., Niskanen & Liimat.	Symbiotic
Cortinarius neofallax Carteret & Reumaux	Symbiotic
Cortinarius niveotraganus Kytöv., Niskanen & Liimat. 2014	Symbiotic
Cortinarius nolaneiformis (Velen.) G. Garnier 1991	Symbiotic
Cortinarius obtusus (Fr.) Fr.	Symbiotic
Cortinarius olivaceofuscus Kühner 1955	Symbiotic
Cortinarius olivaceolamellatus Lebeuf, A.Paul & J.Landry	Symbiotic
Cortinarius pallidibrunneus Niskanen, Kytov. & Liimat.	Symbiotic
Cortinarius paragaudis Fr.	Symbiotic

Cortinarius pardinipes Romagn. 1977	Symbiotic
Cortinarius pholideus (Lilj.) Fr. 1838,	Symbiotic
Cortinarius pilatii Svrček	Symbiotic
Cortinarius pseudobulliardioides Kytöv., Niskanen, Liimat. & Ammirati	Symbiotic
Cortinarius punctatiformis Carteret	Symbiotic
Cortinarius purpurascens Fr.	Symbiotic
Cortinarius rapaceus Fr.	Symbiotic
Cortinarius raphanoides (Pers.) Fr.	Symbiotic
Cortinarius rubrobrunneus Ammirati, Liimat. & Niskanen 2014,	Symbiotic
Cortinarius russulaespermus Carteret	Symbiotic
Cortinarius sanguineus (Wulfen.) Fr.	Symbiotic
Cortinarius scobinaceus Malençon & Bertault	Symbiotic
Cortinarius similis (E. Horak) Peintner, E. Horak, M.M. Moser & Vilgalys 2002	Symbiotic
Cortinarius spisnii Consiglio, D.Antonini & M.Antonini	Symbiotic
Cortinarius subargyronotus Niskanen, Liimat. & Kytöv. 2014,	Symbiotic
Cortinarius subexitiosus Liimat., Niskanen, Kytöv. & Ammirati	Symbiotic
Cortinarius tenuipes （Hongo） Hongo	Symbiotic
Cortinarius tillamookensis Ammirati, Liimat. & Niskanen	Symbiotic
Cortinarius torvus (Fr.) Fr., 1838	Symbiotic
Cortinarius ultimiionophyllus Kytöv., Niskanen & Liimat.	Symbiotic
Cortinarius vaccinochelis Chevassut & Rob.Henry	Symbiotic
Cortinarius violaceonitens (Rob.Henry) Moënne-Locc.	Symbiotic
Cortinarius violaceus (L.) Gray	Symbiotic
Cortinarius viridiflavus Ammirati, Bojantchev, Liimat. & Niskanen	Symbiotic
Cortinarius viridipes M.M.Moser	Symbiotic
Cortinarius xanthochlorus Rob. Henry 1966	Symbiotic
Cortinarius xanthophyllus (Cooke) Rob.Henry.	Symbiotic
Cotylidia carpatica (Pilát) Huijsman	Saprotrophic
Craterellus caeruleofuscus A.H.Sm.	Symbiotic
Craterellus cornucopioides (L.) Pers.	Symbiotic
Craterellus hesleri R.H.Petersen	Symbiotic
Craterellus parvogriseus U.Singh, K.Das & Buyck	Symbiotic
Craterellus sinuosus Fr.	Symbiotic
Crepidotus applanatus (Pers.) P.Kumm.	Saprotrophic
Crepidotus crocophyllus (Berk.) Sacc.	Saprotrophic
Crepidotus malachius Sacc.	Saprotrophic
Crepidotus mollis (Schaeff.) Staude	Saprotrophic
Crepidotus reticulatus T. Bau & Y.P. Ge 2020	Saprotrophic
Crucibulum laeve (Huds.) Kambly, 1936	Saprotrophic

Cruentomycena orientalis Har.Takah. & Taneyama	Saprotrophic
Cudonia circinans (Pers.) Fr. 1849	Saprotrophic
Cudonia lutea (Peck) Sacc. 1885	Saprotrophic
Cuphophyllus colemannianus (A.Bloxam) Bon	Saprotrophic
Cuphophyllus fornicatus (Fr.) Lodge, Padamsee & Vizzini	Saprotrophic
Cuphophyllus pratensis (Pers.) Bon	Saprotrophic
Cuphophyllus virgineus (Wulfen) Kovalenko	Saprotrophic
Cyanoboletus hymenoglutinosus D.Chakraborty, K.Das, A.Baghela, S.K.Singh & Dentinger, 2016	Symbiotic
Cyanosporus bifarius (Spirin) B.K. Cui & Shun Liu 2021	Saprotrophic
Cyanosporus caesius (Schrad.) McGinty 1909	Saprotrophic
Cyanosporus glaucus (Spirin & Miettinen) B.K. Cui & Shun Liu 2021	Saprotrophic
Cyathus lijiangensis T.X. Zhou & R.L. Zhao 2004	Saprotrophic
Cyathus striatus (Huds.) Willd.	Saprotrophic
Cystolepiota sistrata (Fr.) Singer ex Bon & Bellù	Saprotrophic
Dacrymyces australis Lloyd 1920	Saprotrophic
Dacryopinax spathularia (Schwein.) G.W. Martin, 1948	Saprotrophic
Daedaleopsis confragosa (Bolton) J.Schröt.	Saprotrophic
Daedaleopsis tricolor (Bull.) Bondartsev &Singer	Saprotrophic
Daldinia fissa Lloyd	Saprotrophic
Diplomitoporus flavescens (Bres.) Domański	Saprotrophic
Disciseda candida (Schwein.) Lloyd	Saprotrophic
Donadinia sibirica M.Carbone, Agnello, P.Alvarado & Krom	Saprotrophic
Entoloma alboumbonatum Hesler	Saprotrophic
Entoloma aurorae-borealis Noordel., Weholt, Eidissen & Lorås	Saprotrophic
Entoloma byssisedum (Pers.) Donk	Saprotrophic
Entoloma cetratum f. minimosporum	Saprotrophic
Entoloma chalybaeum (Fr.) Noordel.	Saprotrophic
Entoloma clandestinum (Fr.) Noordel.	Saprotrophic
Entoloma conferendum (Britzelm.) Noordel.	Saprotrophic
Entoloma cremeoalbum J.B.Jordal & Noordel.	Saprotrophic
Entoloma cuneatum (Bres.) M.M.Moser	Saprotrophic
Entoloma ekaterinae O.V.Morozova, Noordel., K.Nara, Dima & Brandrud	Saprotrophic
Entoloma eminens Kokkonen	Saprotrophic
Entoloma fuligineocinereum Mešić & Tkalčec	Saprotrophic
Entoloma holmvassdalenense Eidissen, Lorås & Weholt	Saprotrophic
Entoloma insidiosum Noordel.	Saprotrophic
Entoloma lividoalbum (Kühner & Romagn.) Kubička	Saprotrophic
Entoloma longistriatum (Peck) Noordel.	Saprotrophic
Entoloma neglectum (Lasch) Arnolds	Saprotrophic
Entoloma ochromicaceum Noordel. & Liiv	Saprotrophic
Entoloma pallescens (P. Karst.) Noordel. 1979	Saprotrophic

Entoloma politum (Pers.) Noordel.	Saprotrophic
Entoloma prunuloides (Fr.) Quél.	Saprotrophic
Entoloma pulchellum (Hongo) Hongo	Saprotrophic
Entoloma sericeum f. flexipes (J.Favre) E.Horak	Saprotrophic
Entoloma serrulatum (Fr.) Hesler	Saprotrophic
Entoloma undatum (Gillet) M.M.Moser	Saprotrophic
Entoloma xanthochroum (P.D.Orton) Noordel.	Saprotrophic
Exidia glandulosa (Bull.) Fr.	Saprotrophic
Exidia qinghaiensis S.R. Wang & Thorn 2021,	Saprotrophic
Exidia saccharina Fr.	Saprotrophic
Exidia thuretiana (Lév.) Fr.	Saprotrophic
Exidiopsis calcea (Pers.) K.Wells	Saprotrophic
Exidiopsis effusa (Bref. ex Sacc.) Möller	Saprotrophic
Fayodia bisphaerigera (J.E.Lange) Singer	Saprotrophic
Fibroporia citrina (Bernicchia & Ryvarden) Bernicchia & Ryvarden	Saprotrophic
Flagelloscypha minutissima (Burt) Donk	Saprotrophic
Flammulaster pulveraceus E.Horak	Saprotrophic
Fomes formentarius (L.et Fr.) Kickx	Saprotrophic
Fomitopsis fragilis B.K. Cui & M.L. Han	Saprotrophic
Fuscoporia setifer (T. Hatt.) Y.C.Dai	Saprotrophic
Galerina clavata (Velen.) KA%BChner 1935	Saprotrophic
Galerina fasciculata Hongo	Saprotrophic
Galerina fibrillosa A.H.Sm.	Saprotrophic
Galiella amurensis (Lj.N.Vassiljeva) Raitv.	Saprotrophic
Galzinia longibasidia Hallenb.	Saprotrophic
Ganoderma applanatum (Pers.) Pat.	Saprotrophic
Ganoderma australe (Fr.) Pat.	Saprotrophic
Ganoderma leucocontextum T.H.Li, W.Q.Deng, Sheng H.Wu, Dong M.Wang & H.P.Hu	Saprotrophic
Gautieria globispora K.Tao, Ming C.Chang & B.Liu	Symbiotic
Gautieria morchelliformis Vittad.	Symbiotic
Geastrum velutinum Morgan	Symbiotic
Geoglossum barlae Boud.	Saprotrophic
Geoglossum fallax var. subpumilum (S.Imai) S.Imai	Saprotrophic
Geoglossum glabrum f. sphagnophilum (Ehrenb.) J.Favre	Saprotrophic
Geoglossum umbratile var. heterosporum (Mains) Maas Geest.	Saprotrophic
Geopora tenuis (Fuckel) T.Schumach.	Saprotrophic
Geopyxis rehmii Turnau	Saprotrophic
Gliophorus irrigatus (Pers.) A.M.Ainsw. & P.M.Kirk	Saprotrophic
Gliophorus psittacinus (Schaeff.) Herink	Saprotrophic
Gloeophyllum sepiarium (Wulfen) P.Karst.	Saprotrophic
Gloeophyllum striatum (Swartz) Murrill	Saprotrophic
Gloeoporus taxicola (Pers.)Gilb.& Ryvarden	Saprotrophic

Gomphidius glutinosus (Schaeff.) Fr.	Symbiotic
Gomphidius pseudoglutinosus K.Das, Hembrom, A.Parihar & Vizzini	Symbiotic
Gomphus floccosus (Schw.) Singer	Symbiotic
Gomphus orientalis R.H. Petersen & M. Zang	Symbiotic
Guepiniopsis buccina (Pers.) L.L. Kenn. 1959,	Saprotrophic
Gymnopilus aeruginosus (Peck) Singer	Saprotrophic
Gymnopilus bellulus (Peck) Murrill	Saprotrophic
Gymnopilus luteofolius (Peck) Singer	Saprotrophic
Gymnopilus neerlandicus (Huijsman) Contu	Saprotrophic
Gymnopilus sapineus (Fr.) Murrill	Saprotrophic
Gymnopilus subpurpuratus Guzm.-Dáv. & Guzmán 1991	Saprotrophic
Gymnopus aquosus (Bull.) Antonín & Noordel. 1997	Saprotrophic
Gymnopus biformis (Peck) Halling	Saprotrophic
Gymnopus contrarius (Peck) Halling 1997	Saprotrophic
Gymnopus dryophilus JGI SC001DCMIDF	Saprotrophic
Gymnopus hybridus (Kühner & Romagn.) Antonín & Noordel. 1997,	Saprotrophic
Gymnopus subsulphureus (Peck) Murrill	Saprotrophic
Gyromitra infula (Schaeff.et Fr.) Qul.	Saprotrophic
Gyroporus castaneus (Bull.) Quél.	Symbiotic
Gyroporus longicystidiatus Nagas. & Hongo	Symbiotic
Haploporus odorus (Sommerf.) Bondartsev & Singer	Saprotrophic
Harrya chromapes (Frost) Halling, Nuhn, Osmundson & Manfr.Binder, 2012	Symbiotic
Hebeloma alpinum (J. Favre) Bruchet 1970	Symbiotic
Hebeloma cavipes Huijsman 1961	Symbiotic
Hebeloma circinans (Quél.) Sacc.	Symbiotic
Hebeloma nanum Velen. 1940	Symbiotic
Hebeloma plesiocistum Beker, U.Eberh. & Vila	Symbiotic
Hebeloma sordescens Vesterh.	Symbiotic
Hebeloma subconcolor Bruchet	Symbiotic
Hebeloma testaceum (Fr.) Quèl.	Symbiotic
Hebeloma theobrominum Quadr.	Symbiotic
Hebeloma vaccinum Romagn., 1965	Symbiotic
Helvella elastica Bull.	Saprotrophic
Helvella fibrosa (Wallr.) Korf 2008,	Saprotrophic
Helvella floriformis Schaeff.	Saprotrophic
Helvella guttata Q. Zhao & J.R. Lu 2023	Saprotrophic
Helvella lacunosa Afzel.	Saprotrophic
Helvella maculata N.S.Weber	Saprotrophic
Helvella philonotis Dissing	Saprotrophic
Helvellosebacina concrescens (Schwein.) Oberw., Garnica & K.Riess	Symbiotic

Hemimycena albicolor (A.H.Sm.) Elborne	Saprotrophic
Hemimycena angustispora (P.D.Orton) Singer	Saprotrophic
Hemimycena gracilis (Quél.) Singer	Saprotrophic
Hemimycena lacatea (Pers.) Singer	Saprotrophic
Hemimycena mairei (E.-J.Gilbert) Singer	Saprotrophic
Hemimycena ochrogaleata (J.Favre) M.M.Moser	Saprotrophic
Henningsomyces candidus (Pers.) Kuntze	Saprotrophic
Hericium americanum Ginns	Saprotrophic
Heterobasidion insulare (Murrill) Ryvarden 1972	Saprotrophic
Heterobasidion orientale Tokuda, T. Hatt. & Y.C. Dai 2009,	Saprotrophic
Hodophilus hesleri Adamčík, Birkebak & Looney	Saprotrophic
Hohenbuehelia grisea (Peck) Singer	Saprotrophic
Hohenbuehelia longipes (Boud.) M.M.Moser	Saprotrophic
Hohenbuehelia subreniformis (Thorn & G.L. Barron) Thorn 2013	Saprotrophic
Hortiboletus rubellus (Krombh.) Simonini, Vizzini & Gelardi	Symbiotic
Humaria hemisphaerica (F.H.Wigg.) Fuckel	Saprotrophic
Hydnellum caeruleum (Hornem.) P.Karst.	Symbiotic
Hydnellum concrescens (Pers.) Banker	Symbiotic
Hydnellum gracilipes (P.Karst.) P.Karst.	Symbiotic
Hydnellum rubidofuscum Y.H.Mu & H.S.Yuan	Symbiotic
Hydnellum spongiosipes (Peck) Pouzar	Symbiotic
Hydnotrya cerebriformis Harkn.	Symbiotic
Hydropus hymenocephalus (Singer) Redhead	Saprotrophic
Hydropus moserianus Bas	Saprotrophic
Hygrocybe acutoconica (Clem.) Singer	Saprotrophic
Hygrocybe cantharellus (Schwein.) Murrill	Saprotrophic
Hygrocybe ceracea (Sowerby) P.Kumm.	Saprotrophic
Hygrocybe coccineocrenata (P.D.Orton) M.M.Moser	Saprotrophic
Hygrocybe conica (Schaeff.) P.Kumm.	Saprotrophic
Hygrocybe flavescens (Kauffman) Singer	Saprotrophic
Hygrocybe insipida (J.E.Lange) M.M.Moser	Saprotrophic
Hygrocybe irrigata (Pers.) Bon	Saprotrophic
Hygrocybe konradii var. konradii	Saprotrophic
Hygrocybe miniata (Fr.) P.Kumm.	Saprotrophic
Hygrocybe nigrescens (Quél.) Kühner	Saprotrophic
Hygrocybe pratensis (Pers.) Murrill	Saprotrophic
Hygronarius renidens (Fr.) Niskanen & Liimat.	Symbiotic
Hygrophorus cantharellus (Schw.) Fr.	Symbiotic
Hygrophorus coccineus (Schaeff.)Fr.	Symbiotic
Hygrophorus deliciosus C.Q.Wang & T.H.Li	Symbiotic
Hygrophorus discoideus (Pers.) Fr.	Symbiotic
Hygrophorus filavescens (Kauffm.)Smith et Hesler	Symbiotic
Hygrophorus fuscopapillatus C.Q. Wang & T.H. Li 2019	Symbiotic

Hygrophorus imazeki (Hongo) Hongo	Symbiotic
Hygrophorus olivaceoalbus (Fr.) Fr.	Symbiotic
Hygrophorus persicolor Ricek	Symbiotic
Hygrophorus purpurascens (Alb. & Schwein.) Fr.	Symbiotic
Hygrophorus pustulatoides Lebeuf, E.Larss. & Bellanger	Symbiotic
Hygrophorus scabrellus A. Naseer & A.N. Khalid 2019	Symbiotic
Hymenochaete cruenta (Pers.)Donk	Saprotrophic
Hymenochaete sphaericola Lloyd	Saprotrophic
Hymenochaete tenuis Peck	Saprotrophic
Hymenoscyphus immutabilis (Fuckel) Dennis	Saprotrophic
Hyphodontia pachyspora Xue W.Wang & L.W.Zhou	Saprotrophic
Hyphodontia pallidula (Bres.) J.Erikss.	Saprotrophic
Hypholoma capnoides (Fr.) P.Kumm.	Saprotrophic
Hypholoma fasciculare (Huds.) P.Kumm.	Saprotrophic
Hypoxylon rutilum Tul. & C.Tul.	Saprotrophic
Infundibulicybe ellipsospora Z.M. He & Zhu L. Yang 2023	Saprotrophic
Infundibulicybe gibba (Pers.) P. Kumm. 1871	Saprotrophic
Inocybe abdita Bandini & U. Eberh. 2022	Symbiotic
Inocybe abjecta Sacc., 1887	Symbiotic
Inocybe adaequata (Britzelm.) Sacc.	Symbiotic
Inocybe ahmadii Farooqi, Niazi & Khalid	Symbiotic
Inocybe amblyospora Kühner	Symbiotic
Inocybe appendiculata Kühner	Symbiotic
Inocybe auricomella Kühner	Symbiotic
Inocybe blandula Bandini, B.Oertel & U.Eberh.	Symbiotic
Inocybe boreocarelica Kokkonen & Vauras	Symbiotic
Inocybe botaurina Bandini & B.Oertel	Symbiotic
Inocybe bufonia Kokkonen & Vauras	Symbiotic
Inocybe calospora Quél.	Symbiotic
Inocybe casimiri Velen.	Symbiotic
Inocybe catalaunica Singer	Symbiotic
Inocybe cercocarpi Kropp, Matheny & L.J.Hutchison	Symbiotic
Inocybe ceskae Bandini, Esteve-Rav. & B.Oertel	Symbiotic
Inocybe cincinnata (Fr.) Quél.	Symbiotic
Inocybe clandestina Bandini, B.Oertel & U.Eberh.	Symbiotic
Inocybe curvipes P. Karst.	Symbiotic
Inocybe decemgibbosa (Kühner) Vauras	Symbiotic
Inocybe dulcamara (Pers.) P.Kumm.	Symbiotic
Inocybe euganea Giliberto, P.Alvarado & Bizio	Symbiotic
Inocybe filiana Bandini, B.Oertel & U.Eberh.	Symbiotic
Inocybe flavella P.Karst.	Symbiotic
Inocybe flocculosa var. flocculosa	Symbiotic
Inocybe fuscescentipes Kühner	Symbiotic
Inocybe geophylla P.Kumm.	Symbiotic

Inocybe geraniodora J.Favre	Symbiotic
Inocybe glabripes Ricken	Symbiotic
Inocybe godfrinioides Kühner	Symbiotic
Inocybe grammata var. chamaesalicis	Symbiotic
Inocybe grammatoides Esteve-Rav., Pancorbo & E.Rubio	Symbiotic
Inocybe griseoscabrosa (Peck) Earle	Symbiotic
Inocybe involuta Kuyper	Symbiotic
Inocybe ionochlora Romagn.	Symbiotic
Inocybe johannae Kühner	Symbiotic
Inocybe johannis-stanglii Bandini, Esteve-Rav. & G.Moreno	Symbiotic
Inocybe jucunda Bandini, B.Oertel & U.Eberh.	Symbiotic
Inocybe juniperina M.Marchetti, Franchi & Bizio	Symbiotic
Inocybe lacera var. lacera	Symbiotic
Inocybe lanatodisca Kauffman	Symbiotic
Inocybe lanatopurpurea Esteve-Rav. & G.Moreno	Symbiotic
Inocybe lanuginosa (Bull.) P.Kumm.	Symbiotic
Inocybe lapidicola Brugaletta, Consiglio & M.Marchetti	Symbiotic
Inocybe lutescens Velen.	Symbiotic
Inocybe mimica Massee	Symbiotic
Inocybe mixtilis (Britzelm.) Sacc.	Symbiotic
Inocybe montana Kobayasi	Symbiotic
Inocybe muricellata Bres.	Symbiotic
Inocybe nemorosa (R.Heim) Grund & D.E.Stuntz	Symbiotic
Inocybe nitidiuscula (Britzelm.) Lapl., 1894	Symbiotic
Inocybe nivea E.Larss.	Symbiotic
Inocybe obscurobadia (J.Favre) Grund & D.E.Stuntz	Symbiotic
Inocybe occulta Esteve-Rav., Bandini, B.Oertel & G.Moreno	Symbiotic
Inocybe ochroalba Bruyl.	Symbiotic
Inocybe petiginosa (Fr.) Gillet	Symbiotic
Inocybe pholiotinoides Romagn.	Symbiotic
Inocybe plurabellae Bandini, B.Oertel & U.Eberh.	Symbiotic
Inocybe porcorum Vauras & Kokkonen	Symbiotic
Inocybe pseudodestricta Stangl & J. Veselský, 1973	Symbiotic
Inocybe pseudorubens Carteret & Reumaux	Symbiotic
Inocybe pseudoteraturgus Vauras & Kokkonen	Symbiotic
Inocybe purpureobadia Esteve-Rav. & A.Caball.	Symbiotic
Inocybe queletii Konrad	Symbiotic
Inocybe renispora E.Horak	Symbiotic
Inocybe rimosa (Bull.) P.Kumm.	Symbiotic
Inocybe rufoalba Sacc.	Symbiotic
Inocybe salicis-herbaceae Kühner	Symbiotic
Inocybe sambucella G.F.Atk.	Symbiotic
Inocybe splendentoides Bon 1990	Symbiotic
Inocybe suaveolens D.E. Stuntz 1950	Symbiotic

Inocybe sublilacina Matheny & A.Voitk	Symbiotic
Inocybe sublilacina Matheny & A.Voitk	Symbiotic
Inocybe subradiata Murrill	Symbiotic
Inocybe teraturgus M.M.Moser	Symbiotic
Inocybe terrigena (Fr.) Kühner	Symbiotic
Inocybe transitoria (Britzelm.) Sacc.	Symbiotic
Inocybe umbrinodisca Kühner	Symbiotic
Inocybe umbrinofusca Kühner	Symbiotic
Inocybe venerabilis Bandini, B.Oertel & U.Eberh.	Symbiotic
Inocybe vulpinella Bruyl.	Symbiotic
Inocybe xanthomelas Boursier & Kühner	Symbiotic
Inosperma acutofulvum (Bizio & Castellan) Bizio, A.Castellan & Cervini	Symbiotic
Inosperma geraniodorum (J.Favre) Matheny & Esteve-Rav.	Symbiotic
Inosperma maculatum (Boud.) Matheny & Esteve-Rav., 2019 in [Matheny PB et al. (2020)]	Symbiotic
Inosperma rosellicaulare (Grund & D.E.Stuntz) Matheny & Esteve-Rav.	Symbiotic
Ionomidotis fulvotingens (Berk. & M.A. Curtis) E.K. Cash 1939,	Saprotrophic
Irpex lacteus (Fr.) Fr., 1828	Saprotrophic
Jaapia ochroleuca (Bres.) Nannf. & J.Erikss.	Saprotrophic
Kavinia alboviridis (Morgan) Gilb. & Budington	Saprotrophic
Kuehneromyces mutabilis (Schaeff.) Singer & A.H.Sm.	Saprotrophic
Laccaria acanthospora A.W.Wilson & G.M.Muell.	Symbiotic
Laccaria alba Zhu L.Yang & Lan Wang	Symbiotic
Laccaria bicolor (Maire) P.D.Orton	Symbiotic
Laccaria fulvogrisea Popa, Rexer & G.Kost	Symbiotic
Laccaria laccata (Scop. Et Fr.)	Symbiotic
Laccaria moshuijun Popa & Zhu L. Yang 2017	Symbiotic
Laccaria negrimarginata A.W.Wilson & G.M.Muell.	Symbiotic
Laccaria picinus Fr.	Symbiotic
Laccaria proxima (Boud.) Pat.	Symbiotic
Laccaria pumila Fayod	Symbiotic
Laccaria salmonicolor A.W.Wilson & G.M.Muell.	Symbiotic
Laccaria tortilis (Bolton) Cooke	Symbiotic
Lachnum pulverulentum (Lib.) P.Karst.	Saprotrophic
Lachnum virgineum (Batsch) P.Karst.	Saprotrophic
Lacrymaria lacrymabunda (Bull.) Pat.	Saprotrophic
Lactarius alnicola A.H.Sm.	Symbiotic
Lactarius alpinihirtipes X.H.Wang	Symbiotic
Lactarius aquizonatus Kytöv.	Symbiotic
Lactarius aurantiacus (Pers.) Gray	Symbiotic
Lactarius aurantiosordidus Nuytinck & S.L.Mill.	Symbiotic

Lactarius badiosanguineus Kühner & Romagn.	Symbiotic
Lactarius camphorates (Bull.) Fr.	Symbiotic
Lactarius deterrimus Gröger	Symbiotic
Lactarius fulvihirtipes X.H.Wang	Symbiotic
Lactarius glyciosmus (Fr.) Fr.	Symbiotic
Lactarius hirtipes J.Z.Ying	Symbiotic
Lactarius hysginus Fr.	Symbiotic
Lactarius indigo (Schw.) Fr.	Symbiotic
Lactarius pallidizonatus X.H.Wang	Symbiotic
Lactarius picinus Fr.	Symbiotic
Lactarius piperatus (L.) Pers.	Symbiotic
Lactarius pseudofragilis X.H. Wang 2017	Symbiotic
Lactarius pseudohatsudake X.H.Wang	Symbiotic
Lactarius pterosporus Romagn., 1949	Symbiotic
Lactarius pubescens Fr.	Symbiotic
Lactarius purpureus R.Heim	Symbiotic
Lactarius rufus (Scop.) Fr.	Symbiotic
Lactarius scrobiculatus var. pubescens	Symbiotic
Lactarius sphagneti (Fr.) Neuhoff	Symbiotic
Lactarius spinosulus Quél. & Le Bret.	Symbiotic
Lactarius subvellereus Peck	Symbiotic
Lactarius torminosus (Schaeff.) Pers.	Symbiotic
Lactarius trivialis (Fr.) Fr. 1838	Symbiotic
Lactifluus pilosus (Verbeken, H.T.Le & Lumyong) Verbeken	Symbiotic
Leccinum extremioroentale (Li.N. Vassiljeva) Singer	Symbiotic
Leccinum roseotinctum Watling 1969	Symbiotic
Leccinum rugosiceps (Peck) Singer 1945,	Symbiotic
Leccinum scabrum (Bull.et Fr.) Gray	Symbiotic
Leccinum schistophilum Bon 1981	Symbiotic
Legaliana badia (Pers.) Van Vooren 2020	Saprotrophic
Lentinellus cochleatus (Pers.) P.Karst.	Saprotrophic
Lentinellus flabelliformis (Bolton) S. Ito 1959	Saprotrophic
Lentinellus subargillaceus (Kauffman) R.H.Petersen	Saprotrophic
Lentinellus sublineolatus R.H.Petersen	Saprotrophic
Lentinellus ursinus (Fr.) Kühner	Saprotrophic
Lentinus arcularius (Batsch) Zmitr.	Saprotrophic
Lentinus sajor-caju Fr.	Saprotrophic
Lentinus suavissimus Fr. 1836,	Saprotrophic
Leotia lubrica (Scop.) Pers.	Saprotrophic
Leotia viscosa Fr. 1822	Saprotrophic
Lepiota castanea var. vinosobrunnea J.Aug.Schmitt	Saprotrophic
Lepiota coloratipes Vizzini, J.F.Liang, Jančovič. & Zhu L.Yang	Saprotrophic
Lepiota cortinarius J.E.Lange	Saprotrophic

Lepiota cristata (Bolton) P.Kumm.	Saprotrophic
Lepiota rufipes f. phaeophylla Bon	Saprotrophic
Lepista nuda (Bull.) Cooke	Saprotrophic
Lepista panaeolus (Fr.) P.Karst.	Saprotrophic
Lepista sordida (Schumach.) Singer	Saprotrophic
Leucoagaricus lahorensiformis S.Hussain, H.Ahmad, Afshan & Khalid	Saprotrophic
Leucoagaricus nympharum (Kalchbr.) Bon	Saprotrophic
Leucocortinarius bulbiger (Alb. & Schwein.) Singer 1945	Symbiotic
Leucophleps spinispora Fogel	Symbiotic
Limacella ochraceolutea P.D. Orton	Saprotrophic
Lycoperdon curtisii Berk., 1873	Saprotrophic
Lycoperdon dermoxanthum Vittad.	Saprotrophic
Lycoperdon ericaeum Bonord.	Saprotrophic
Lycoperdon excipuliforme (Scop.) Pers.	Saprotrophic
Lycoperdon mammiforme Pers.	Saprotrophic
Lycoperdon molle Pers., 1801	Saprotrophic
Lycoperdon niveum Kreisel 1969	Saprotrophic
Lycoperdon perlatum Pers.	Saprotrophic
Lycoperdon pratense Pers.	Saprotrophic
Lycoperdon pyriforme Schaeff.	Saprotrophic
Lycoperdon rimulatum Peck	Saprotrophic
Lycoperdon rupicola Jeppson, E. Larss. & M.P. Martín 2012	Saprotrophic
Lycoperdon subumbrinum Jeppson & E.Larss.	Saprotrophic
Lycoperdon umbrinum Pers.	Saprotrophic
Lycoperdon utriforme Bull.	Saprotrophic
Lyophyllum conglobatum var. albidopallidum Banares & Bon, 2008	Saprotrophic
Lyophyllum fumosum (Pers.) P.D.Orton	Saprotrophic
Lyophyllum turcicum Sesli, Vizzini & Contu	Saprotrophic
Macowanites chlorinosmus A.H.Sm. & Trappe	Symbiotic
Macrocystidia cucumis (Pers.) Joss.	Saprotrophic
Mallocybe crassivelata Ferisin, Bizio, Esteve-Rav., Vizzini & Dovana	Symbiotic
Mallocybe malenconii (R.Heim) Matheny & Esteve-Rav.	Symbiotic
Mallocybe siciliana (Brugaletta, Consiglio & M.Marchetti) Brugaletta, Consiglio & M.Marchetti	Symbiotic
Mallocybe subdecurrens (Ellis & Everh.) Matheny & Esteve-Rav.	Symbiotic
Mallocybe terrigena (Fr.) Matheny, Vizzini & Esteve-Rav.	Symbiotic
Marasmiellus quercinus Singer	Saprotrophic
Marasmiellus troyanus (Murrill) Dennis	Saprotrophic
Marasmius corrugatus (Pat.) Sacc. & P.Syd.	Saprotrophic

Marasmius cupreostipes Wannathes, Desjardin & Lumyong 2009	Saprotrophic
Marasmius maximus Hongo	Saprotrophic
Melanoleuca brevipes (Bull.) Pat	Saprotrophic
Melanoleuca verrucipes (Fr.) Singer 1939	Saprotrophic
Melanophyllum haematospermum (Bull.) Kreisel	Saprotrophic
Miladina lecithina (Cooke) Svrček	Saprotrophic
Mitrula brevispora Zheng Wang	Saprotrophic
Morchella norvegiensis Jacquet., 1985	Saprotrophic
Mycena abramsii (Murrill) Murrill	Saprotrophic
Mycena acicula (Schaeff.) P.Kumm.	Saprotrophic
Mycena adnexa T. Bau & Q.Na	Saprotrophic
Mycena amicta (Fr.) Quél.	Saprotrophic
Mycena haematopus (Pers.) P. Kumm. 1871	Saprotrophic
Mycena laevigata (Lasch) Gillet 1876	Saprotrophic
Mycena leaiana (Berk.) Sacc.	Saprotrophic
Mycena metata (Fr.) P.Kumm.	Saprotrophic
Mycena pearsoniana Dennis, 1955	Saprotrophic
Mycena pura (Pers.) P.Kumm.	Saprotrophic
Mycena rebaudengoi Robich	Saprotrophic
Mycena rosella (Fr.) P.Kumm.	Saprotrophic
Mycena sanguinolenta (Alb. & Schwein.) P. Kumm., 1871	Saprotrophic
Mycena seminau A.L.C. Chew & Desjardin 2014	Saprotrophic
Mycena stylobates (Pers.) P.Kumm.	Saprotrophic
Mycena viridimarginata P.Karst.	Saprotrophic
Mycenella bryophila (Voglino) Singer	Saprotrophic
Mycenella lasiosperma (Bres.) Locq.	Saprotrophic
Mycetinis salalis (Desjardin & Redhead) Redhead 2012	Saprotrophic
Mycetinis scorodonius (Fr.) A.W.Wilson & Desjardin	Saprotrophic
Mycopan scabripes (Murrill) Redhead, Moncalvo & Vilgalys	Saprotrophic
Naematelia aurantialba (Bandoni & M. Zang) Millanes & Wedin	Facultatively parasitic/saprotrophic
Naucoria bohemica Velen.	Saprotrophic
Naucoria paludosa Peck	Saprotrophic
Neoboletus sanguineus (G. Wu & Zhu L. Yang) N.K. Zeng et al.	Symbiotic
Neofavolus alveolaris (DC.) Sotome & T. Hatt. 2012	Saprotrophic
Neofavolus americanus J.H. Xing, J.L. Zhou & B.K. Cui 2020	Saprotrophic
Neofavolus cremeoalbidus Sotome & T. Hatt. 2012,	Saprotrophic
Notholepista fistulosa Z.M. He & Zhu L. Yang 2022	Saprotrophic
Octaviania mortae Orihara	Symbiotic
Omphalina ericetorum (Pers.) M.Lange	Saprotrophic
Ossicaulis yunnanensis L.P. Tang, N.K. Zeng & S.D. Yang 2017	Saprotrophic

Otidea alutacea (Pers.) Massee	Saprotrophic
Otidea bufonia (Pers.) Boud.	Saprotrophic
Otidea caeruleopruinosa Harmaja	Saprotrophic
Otidea cochleata (L.) Fuckel	Saprotrophic
Otidea olivaceobrunnea Harmaja 2009,	Saprotrophic
Pachylepyrium nubicola Singer	Saprotrophic
Pallidohirschioporus biformis (Fr.) Y.C. Dai, Yuan Yuan & Meng Zhou 2023	Saprotrophic
Panaeolus alcis M.M.Moser	Saprotrophic
Panaeolus campanulatus (L.)Quel	Saprotrophic
Panaeolus fimicola (Pers.) Gillet	Saprotrophic
Panaeolus sphinctrinus (Fr.) QuA%A9l. 1872	Saprotrophic
Panaeolus uliginosus Jul.Schäff.	Saprotrophic
Panellus stipticus (Bull.) P. Karst. 1879	Saprotrophic
Panus conchatus （Bull.）Fr.	Saprotrophic
Panus lecomtei (Fr.) Corner 1981	Saprotrophic
Panus subfasciatus Thongbai, Karun., C. Richt. & K.D. Hyde 2017	Saprotrophic
Parasola plicatilis (Curtis) Redhead, Vilgalys & Hopple	Saprotrophic
Parasola plicatilis-similis L.Nagy, Szarkándi & Dima	Saprotrophic
Paxillus involutus (Batsch) Fr.	Symbiotic
Paxillus obscurisporus C.Hahn	Symbiotic
Peniophora cinerea (Pers.) Cooke	Saprotrophic
Peniophora incarnata (Pers.) P.Karst.	Saprotrophic
Perenniporia fraxinea (Bull.) Ryvarden	Saprotrophic
Perenniporia tibetica B.K. Cui&C.L. Zhao	Saprotrophic
Pezicula melanigena (T.Kowalski & Halmschl.) P.R.Johnst.	Facultatively parasitic/saprotrophic
Peziza alaskana E.K.Cash	Saprotrophic
Peziza badia var. terrestris Alb. & Schwein.	Saprotrophic
Peziza depressa var. applanata (Hedw.) Pers.	Saprotrophic
Peziza fimeti (Fuckel) E.C.Hansen	Saprotrophic
Peziza howsei Boud. ex Donadini	Saprotrophic
Peziza limnaea Maas Geest.	Saprotrophic
Peziza michelii (Boud.) Dennis	Saprotrophic
Peziza ninguis Donadini & Trimbach	Saprotrophic
Peziza nivalis (R.Heim & L.Rémy) M.M.Moser	Saprotrophic
Peziza ostracoderma Korf	Saprotrophic
Peziza saniosa Schrad.	Saprotrophic
Peziza succosa Berk.	Saprotrophic
Phaeolepiota aurea (Bull.) R. Maire ex Konrad & Maubl. 1928	Saprotrophic
Phaeotremella fagi (Middelhoven & Scorzetti) Yurkov & Boekhout	Parasitic
Phaeotremella frondosa (Fr.) Spirin & Malysheva 2018	Parasitic
Phaeotremella fuscosuccinea (Chee J.Chen) Spirin & Yurkov	Parasitic

Phaeotremella mycophaga (G.W.Martin) Millanes & Wedin	Parasitic
Phallus impudicus L.	Saprotrophic
Phallus mengsongensis H.Li Li, L.Ye, P.E.Mortimer, J.C.Xu & K.D.Hyde	Saprotrophic
Phanerochaete viticola (Schwein.) Parmasto	Saprotrophic
Phellinus tuberculosus (Baumg.) Niemela 1982	Saprotrophic
Phellodon atroardesiacus B.K.Cui & C.G.Song	Symbiotic
Phellodon melaleucus (Sw. ex Fr.) P.Karst.	Symbiotic
Phellodon niger (Fr.) P.Karst.	Symbiotic
Phellodon stramineus B.K.Cui & C.G.Song	Symbiotic
Phellodon yunnanensis B.K.Cui & C.G.Song	Symbiotic
Phlebia acerina Peck	Saprotrophic
Phlebia nantahaliensis Nakasone & Burds.	Saprotrophic
Phlebia nitidula (P.Karst.) Ryvarden	Saprotrophic
Phlebia tremellosa (Schrad.) Nakasone & Burds.	Saprotrophic
Phlegmacium caesiocolor (Kytöv., Liimat. & Niskanen) Niskanen & Liimat.	Symbiotic
Pholiota alnicola (Fr.) Singer	Saprotrophic
Pholiota brunnescens A.H.Sm. & Hesler	Saprotrophic
Pholiota fusu (Batsch. Et Fr.)	Saprotrophic
Pholiota gummosa (Lasch) Singer	Saprotrophic
Pholiota humii A.H. Sm. & Hesler 1968	Saprotrophic
Pholiota lenta (Pers.) Singer	Saprotrophic
Pholiota lignicola (Peck) Jacobsson 1989	Saprotrophic
Pholiota lubrica (Pers.) Singer	Saprotrophic
Pholiota malakandensis Z. Ullah, Jabeen, H. Bashir & Khalid 2021,	Saprotrophic
Pholiota multicingulata E.Horak	Saprotrophic
Pholiota spumosa (Fr.) Singer	Saprotrophic
Pholiota squarrosa (Vahl) P.Kumm.	Saprotrophic
Phyllotopsis nidulans (Pers.) Singer 1936	Saprotrophic
Physisporinus crocatus (Pat.) F.Wu, Jia J.Chen & Y.C.Dai	Saprotrophic
Physisporinus eminens (Y.C. Dai) F. Wu,Jia J.Chen & Y.C. Dai	Saprotrophic
Piptoporus betulinus (Bull.) P.Karst.	Saprotrophic
Pistillaria petasitis S. Imai	Saprotrophic
Plectania melastoma (Sowerby) Fuckel	Saprotrophic
Plectania rhytidia (Berk.) Nannf. & Korf	Saprotrophic
Plectania zugazae Calonge & Alb.García	Saprotrophic
Pleurotua cornucopiae (Pual.et Pers.) Roll.	Saprotrophic
Pleurotus calyptratus (Lindblad.) Sacc.	Saprotrophic
Pleurotus cornucopiae (Pual. Et Pers.) Roll.	Saprotrophic
Pleurotus placentodes (Berk.) Sacc. 1887	Saprotrophic
Pleurotus spodoleucus Fr.	Saprotrophic

Plicaria carbonaria Fuckel	Saprotrophic
Pluteus brunneidiscus Murrill 1917,	Saprotrophic
Pluteus cervinus (Schaeff.) P.Kumm.	Saprotrophic
Pluteus cinereofuscus J.E.Lange	Saprotrophic
Pluteus hongoi Singer 1989,	Saprotrophic
Pluteus nothopellitus Justo & M.L.Castro	Saprotrophic
Pluteus pouzarianus Singer	Saprotrophic
Pluteus semibulbosus (Lasch) Quél.	Saprotrophic
Pluteus terricola E.Horak	Saprotrophic
Polyozellus badjelanndanus (Svantesson) Svantesson & Kõljalg	Symbiotic
Polyozellus flavovirens (Höhn. & Litsch.) Svantesson & Kõljalg	Symbiotic
Polyozellus medius (Svantesson & Kõljalg) Svantesson & Kõljalg	Symbiotic
Polyozellus mucidulus (P.Karst.) Svantesson & Kõljalg	Symbiotic
Polyozellus rhizopunctatus (E.C.Martini & Hentic) Svantesson & Kõljalg	Symbiotic
Polyozellus sciastrus (Svantesson & Kõljalg) Svantesson & Kõljalg	Symbiotic
Polyozellus tristis (P.Karst.) Svantesson & Kõljalg	Symbiotic
Polyozellus umbrinus (Fr.) Svantesson & Kõljalg	Symbiotic
Polyporus arcularius (Batsch) Fr.	Saprotrophic
Polyporus cuticulatus Y.C. Dai et al.	Saprotrophic
Polyporus mori (Pollini) Fr.	Saprotrophic
Polyporus tuberaster (Jacq. ex Pers.) Fr. 1821	Saprotrophic
Polyporus umbellatus (Pers.) Fr.	Saprotrophic
Polyporus varius (Pers.) Fr. 1821	Saprotrophic
Polystictus xanthopus Fr.	Saprotrophic
Porodaedalea cancriformans (M.J.Larsen, Lombard & Aho) T.Wagner & M.Fisch.	Saprotrophic
Postia balsamea (Peck) Jülich	Saprotrophic
Postia caesia (Schrad.) P.Karst.	Saprotrophic
Postia fragilis (Fr.) Jülich	Saprotrophic
Postia hirsuta L.L. Shen & B.K. Cui 2014	Saprotrophic
Postia ptychogaster (F.Ludw.) Vesterh.	Saprotrophic
Postia rennyi (Berk. & Broome) Rajchenb.	Saprotrophic
Postia sericeomollis (Romell) Jülich	Saprotrophic
Postia subcaesia (A.David) Jülich	Saprotrophic
Protoglossum niveum (Vittad.) T.W.May	Saprotrophic
Protomerulius brachysporus (Luck-Allen) Spirin & Malysheva	Saprotrophic
Protostropharia semiglobata (Batsch) Redhead, Moncalvo & Vilgalys, 2013	Saprotrophic
Psathyrella bipellis (Quelet) A.H. Smith	Saprotrophic

Psathyrella candolleana (Fr.) Maire, 1937	Saprotrophic
Psathyrella corrugis (Pers.) Konrad & Maubl.	Saprotrophic
Psathyrella pygmaea (Bull.) Singer	Saprotrophic
Psathyrella tenuicula (P.Karst.) Örstadius & Huhtinen	Saprotrophic
Psathyrella violaceopallens Contu	Saprotrophic
Pseudaleuria fibrillosa (Massee) J.Moravec	Saprotrophic
Pseudaleuria quinaultiana Lusk	Saprotrophic
Pseudolyophyllum macrobasidium Z.M. He & Zhu L. Yang 2023	Saprotrophic
Pseudombrophila hepatica (Batsch) Brumm.	Saprotrophic
Pseudoplectania nigrella (Pers.) Fuckel	Saprotrophic
Pseudosperma bulbosissimum (Kühner) Matheny & Esteve-Rav. 2019	Symbiotic
Pseudosperma rimosum (Bull.) Matheny & Esteve-Rav.	Symbiotic
Pseudosperma sororium (Kauffman) Matheny & Esteve-Rav. 2019	Symbiotic
Pseudotomentella atrofusca M.J.Larsen	Symbiotic
Pseudotomentella mucidula (P.Karst.) Svrček	Symbiotic
Psilocybe caerulipes (Peck) Sacc.	Saprotrophic
Psilocybe coprophila (Bull.et Fr.) Kummer	Saprotrophic
Pulvinula carbonaria (Fuckel) Boud.	Saprotrophic
Pycnoporus cinnabarinus (Jacq.) P.Karst.	Saprotrophic
Pycnoporus sanguineus (L.) Murrill	Saprotrophic
Ramaria acrisiccescens Marr & D.E. Stuntz 1974	Symbiotic
Ramaria distinctissima R.H. Petersen & M. Zang	Symbiotic
Ramaria hemirubella Petersen et Zang	Symbiotic
Ramariopsis avellaneo-inversa R.H.Petersen	Saprotrophic
Ramariopsis crocea (Pers.) Corner	Saprotrophic
Ramariopsis flavescens R.H.Petersen	Saprotrophic
Ramariopsis kunzei (Fr.) Corner	Saprotrophic
Rectipilus davidii (D.A.Reid) Agerer	Saprotrophic
Resupinatus applicatus (Batsch) Gray	Saprotrophic
Rhizopogon evadens A.H.Sm.	Symbiotic
Rhizopogon jiyaozi Lin Li & Shu H.Li	Symbiotic
Rhizopogon roseolus (Corda) Th.Fr.	Symbiotic
Rhodocollybia butyracea (Bull.) Lennox	Saprotrophic
Rhodocybe griseonigrella (Vila, Contu, F.Caball. & Ribes) Vizzini, Vila, Picillo & Contu	Saprotrophic
Roridomyces roridus (Fr.) Rexer 1994	Saprotrophic
Roseodiscus subcarneus (Sacc.) Baral	Saprotrophic
Russula acrifolia Romagn.	Symbiotic
Russula adusta (Pers.) Fr.	Symbiotic
Russula aeruginea Lindblad ex Fr.	Symbiotic
Russula anthracina Romagn., 1962	Symbiotic

Russula atroglauca Einhell.	Symbiotic
Russula atropurpurea (Krombh.) Britzelm.	Symbiotic
Russula aurantioflammans Ruots., Sarnari & Vauras	Symbiotic
Russula azurea Bres.	Symbiotic
Russula brevipes Peck	Symbiotic
Russula brunneola Burl.	Symbiotic
Russula candida (Tul. & C.Tul.) J.M.Vidal	Symbiotic
Russula cerolens Shaffer 1972	Symbiotic
Russula cessans A.Pearson	Symbiotic
Russula chiui G.J. Li & H.A. Wen 2015	Symbiotic
Russula chlorineolens Trappe & T.F.Elliott	Symbiotic
Russula chloroides (Krombh.) Bres.	Symbiotic
Russula crustosa Peck, 1887	Symbiotic
Russula cuprea Krombh.	Symbiotic
Russula curtipes F.H. Møller & Jul. Schäff. 1935	Symbiotic
Russula cyanoxantha (Schaeff.) Fr.	Symbiotic
Russula delica Fr.	Symbiotic
Russula depallens (Pers.) Fr.	Symbiotic
Russula emetica (Schaeff.) Pers. 1796	Symbiotic
Russula exalbicans (Pers.) Melzer & Zvára	Symbiotic
Russula farinipes Romell.	Symbiotic
Russula foetens Pers., 1796	Symbiotic
Russula globispora (J.Blum) Bon	Symbiotic
Russula gracilis Burl.	Symbiotic
Russula gracillima Jul.Schäff.	Symbiotic
Russula helios Malençon ex Sarnari	Symbiotic
Russula heterochroa Kühner	Symbiotic
Russula indocatillus A.Ghosh, K.Das & R.P.Bhatt	Symbiotic
Russula innocua (Singer) Romagn. ex Bon	Symbiotic
Russula lakhanpalii A. Ghosh, K. Das & R.P. Bhatt 2019	Symbiotic
Russula levyana Murrill	Symbiotic
Russula lutea (Huds.) Gray 1821	Symbiotic
Russula nigricans Fr.	Symbiotic
Russula nitida (Pers.) Fr.	Symbiotic
Russula nobilis Velen.	Symbiotic
Russula odorata Romagn.	Symbiotic
Russula olivacea Pers., 1796	Symbiotic
Russula paludosa Britzelm.	Symbiotic
Russula pseudochamaeleontina Trendel	Symbiotic
Russula pseudopectinatoides G.J.Li & H.A.Wen	Symbiotic
Russula puellaris Fr.	Symbiotic
Russula pulchra Burl., 1918	Symbiotic
Russula punctata var. seperina (Dupain) Melzer & Zvára	Symbiotic
Russula punctata var. seperina (Dupain) Melzer & Zvára	Symbiotic

Russula punicea W.F.Chiu	Symbiotic
Russula purpureofusca Kühner	Symbiotic
Russula queletii f. albocitrina Barbier	Symbiotic
Russula risigallina (Batsch) Sacc.	Symbiotic
Russula romellii Maire	Symbiotic
Russula roseonigra Pidlich-Aigner	Symbiotic
Russula roseopileata McNabb	Symbiotic
Russula rubra (Krombh.) Bres.	Symbiotic
Russula saliceticola (Singer) Kühner ex Knudsen & T.Borgen	Symbiotic
Russula sanguinea Fr.	Symbiotic
Russula sapinea Sarnari	Symbiotic
Russula sphagnophila Kauffman, 1909	Symbiotic
Russula subnigricans Hongo	Symbiotic
Russula subrubescens Murrill	Symbiotic
Russula turci var. gilva Einhell.	Symbiotic
Russula umerensis McNabb 1973,	Symbiotic
Russula velenovskyi Melzer & Zvára	Symbiotic
Russula vesca Fr.	Symbiotic
Russula vidalii Trappe & T.F.Elliott	Symbiotic
Russula xerampelina var. xerampelina	Symbiotic
Sarcodon coactus Y.H.Mu & H.S.Yuan	Symbiotic
Sarcodon glaucopus Maas Geest. & Nannf.	Symbiotic
Sarcodon imbricatus (L.) P.Karst.	Symbiotic
Sarcodon leucopus (Pers.) Maas Geest. & Nannf.	Symbiotic
Sarcodon scabrosus(Fr.) P. Karst.	Symbiotic
Sarcodon violascens (Alb.et Schw.:Fr.) Quel.	Symbiotic
Sarcoporia polyspora P.Karst.	Saprotrophic
Schizophyllum commune 4.8 streak	Saprotrophic
Sclerodema cepa Pers.	Symbiotic
Scleroderma areolatum Ehrenb. 1818	Symbiotic
Scleroderma bovista Fr.	Symbiotic
Scutellinia scutellata (L.) Lambotte	Saprotrophic
Sebacina candida L.S.Olive	Symbiotic
Sebacina dimitica Oberw.	Symbiotic
Sebacina epigaea (Berk. & Broome) Bourdot & Galzin	Symbiotic
Sebacina incrustans (Pers.) Tul. & C.Tul.	Symbiotic
Sepultariella semi-immersa (P.Karst) Kutorga, 2000	Saprotrophic
Serpula himantioides (Fr.) P.Karst.	Saprotrophic
Simocybe haustellaris (Fr.) Watling	Saprotrophic
Sistotrema confluens Pers.	Saprotrophic
Sistotrema hypogaeum Warcup & P.H.B.Talbot, 1962	Saprotrophic
Sistotrema luteoviride Kotir. & K.H.Larss.	Saprotrophic
Sistotrema muscicola (Pers.) S.Lundell	Saprotrophic
Sistotrema octosporum (J.Schröt. ex Höhn. & Litsch.) Hallenb.	Saprotrophic

Sistotrema sernanderi (Litsch.) Donk	Saprotrophic
Sowerbyella rhenana (Fuckel) J.Moravec	Saprotrophic
Sparassis crispa (Wulfen) Fr.	Symbiotic
Spathularia flavida Persoon, 1797	Saprotrophic
Spodocybe rugosiceps Z.M. He & Zhu L. Yang 2021	Saprotrophic
Steccherinum bourdotii Saliba & A.David	Saprotrophic
Steccherinum laeticolor (Berk. & M.A.Curtis) Banker	Saprotrophic
Steccherinum ochraceum (Pers. ex J.F.Gmel.) Gray	Saprotrophic
Stereopsis vitellina (S.Lundell) D.A.Reid	Saprotrophic
Stereum armeniacum Boidin & Gilles	Saprotrophic
Stereum fasciatum Schw.	Saprotrophic
Stereum hirsutum (Willd.) Pers.	Saprotrophic
Stereum ochraceoflavum (Schwein.) Sacc., 1888	Saprotrophic
Stereum sanguinolentum (Alb. & Schwein.) Fr. 1838	Saprotrophic
Strobilurus occidentalis V.L. Wells & Kempton 1971,	Saprotrophic
Stropharia lignicola E.J. Tian 2021	Saprotrophic
Suillus alpinus X.F. Shi & P.G. Liu 2016,	Symbiotic
Suillus cavipes (Klotzsch) A.H.Sm. & Thiers	Symbiotic
Suillus luteus (L.) Roussel	Symbiotic
Suillus viscidus (L.) Roussel	Symbiotic
Tapinella panuoides (Fr.) E.-J.Gilbert	Saprotrophic
Tarzetta catinus (Holmsk.) Korf & J.K. Rogers 1971	Saprotrophic
Tarzetta confusa F.M. Yu, S. Wang, Q. Zhao & K.D. Hyde 2021,	Saprotrophic
Tarzetta linzhiensis F.M. Yu, S. Wang, Q. Zhao & K.D. Hyde 2021	Saprotrophic
Tephrocybe fibrosipes Métrod ex Bon 1993	Saprotrophic
Tephrocybe ozes (Fr.) Bon 1995,	Saprotrophic
Thanatephorus ochraceus (Massee) P.Roberts	Symbiotic
Thaxterogaster alboamarescens (Kytöv., Niskanen & Liimat.) Niskanen & Liimat.	Symbiotic
Thaxterogaster mendax (Bidaud, Mahiques & Reumaux) Niskanen & Liimat.	Symbiotic
Thaxterogaster talimultiformis (Kytöv., Liimat., Niskanen, A.F.S.Taylor & Sesli) Niskanen & Liimat.	Symbiotic
Thaxterogaster urbiculus (Soop) Niskanen & Liimat. 2022	Symbiotic
Thelephora anthocephala (Bull.) Fr.	Symbiotic
Thelephora atra Weinm.	Symbiotic
Thelephora caryophyllea (Schaeff.) Pers.	Symbiotic
Thelephora palmata (Scop.) Fr.	Symbiotic
Thelephora scissilis Burt	Symbiotic
Thelephora terrestris Ehrh. ex Fr.	Symbiotic
Tolypocladium inegoense (Kobayasi) C.A.Quandt, Kepler & Spatafora	Parasitic

Tolypocladium inflatum W.Gams	Parasitic
Tolypocladium inusitaticapitatum F.M. Yu, Q. Zhao & K.D. Hyde 2021	Parasitic
Tolypocladium ophioglossoides (J.F.Gmel.) C.A.Quandt, Kepler & Spatafora	Parasitic
Tomentella badia (Link) Stalpers	Symbiotic
Tomentella badioincrustata Yorou & Paroll	Symbiotic
Tomentella bryophila (Pers.) M.J.Larsen	Symbiotic
Tomentella coerulea Höhn. & Litsch.	Symbiotic
Tomentella conclusa H.S.Yuan, X.Lu & Y.C.Dai	Symbiotic
Tomentella ellisii (Sacc.) Jülich & Stalpers	Symbiotic
Tomentella ferruginea (Pers.) Pat.	Symbiotic
Tomentella fuscocinerea (Pers.) Donk	Symbiotic
Tomentella fuscocrustosa H.S.Yuan, X.Lu & Y.C.Dai	Symbiotic
Tomentella griseomarginata H.S.Yuan, X.Lu & Y.C.Dai	Symbiotic
Tomentella lapida (Pers.) Stalpers	Symbiotic
Tomentella lateritia Pat.	Symbiotic
Tomentella lilacinogrisea Wakef.	Symbiotic
Tomentella pallidocastanea X.Lu, Y.H.Mu & H.S.Yuan	Symbiotic
Tomentella pallidomarginata H.S.Yuan, X.Lu & Y.C.Dai	Symbiotic
Tomentella pilosa (Burt) Bourdot & Galzin	Symbiotic
Tomentella spinosispora Čížek	Symbiotic
Tomentella stuposa (Link) Stalpers	Symbiotic
Tomentella subclavigera Litsch.	Symbiotic
Tomentella sublilacina (Ellis & Holw.) Wakef.	Symbiotic
Tomentella subtestacea (Bourdot & Galzin) Svrček	Symbiotic
Tomentella tenuirhizomorpha X.Lu, Y.H.Mu & H.S.Yuan	Symbiotic
Tomentella terrestris (Berk. & Broome) M.J.Larsen	Symbiotic
Tomentella viridibasidia Svantesson	Symbiotic
Tomentella viridula (Bourdot & Galzin) Svrček	Symbiotic
Tomentellopsis pulchella Kõljalg & Bernicchia	Symbiotic
Tomentellopsis rosannae Kuhar & Gresl.	Symbiotic
Trametes cinnabarina BRFM137	Saprotrophic
Trametes cubensis (Mont.) Sacc.	Saprotrophic
Trametes gibbosa (Pers.) Fr.	Saprotrophic
Trametes hirsuta (Wulfen) Pilat	Saprotrophic
Trametes ochracea (Pers.) Gilb.& Ryvarden	Saprotrophic
Trametes trogii Berk.	Saprotrophic
Trametes versicolor (L.) Lloyd	Saprotrophic
Trappea darkeri (Zeller) Castellano	Symbiotic
Trechispora caucasica (Parmasto) Liberta	Saprotrophic
Trechispora cohaerens (Schwein.) Jülich & Stalpers	Saprotrophic
Trechispora hymenocystis (Berk. & Broome) K.H.Larss.	Saprotrophic
Trechispora invisitata (H.S.Jacks.) Liberta	Saprotrophic

Trechispora microspora (P.Karst.) Liberta	Saprotrophic
Trechispora mollusca (Pers.) Liberta	Saprotrophic
Trechispora stevensonii (Berk. & Broome) K.H.Larss.	Saprotrophic
Trechispora verruculosa (G.Cunn.) K.H.Larss.	Saprotrophic
Tremella aurantialba Bandoni & M. Zang 1990,	Facultatively parasitic/saprotrophic
Tremella foliacea Pers.	Parasitic
Tremellodendron schweinitzii (Peck) G.F.Atk.	Symbiotic
Tremellodendropsis tuberosa (Grev.) D.A.Crawford	Saprotrophic
Trichaptum abietinum (Pers. ex J.F.Gmel.) Ryvarden	Saprotrophic
Trichapum fuscoviolaceum (Ehrenb.) Ryvarden	Saprotrophic
Tricharina ochroleuca (Sacc.) Eckblad	Saprotrophic
Tricharina praecox (P.Karst.) Dennis	Saprotrophic
Trichoderma americanum (Canham) Jaklitsch & Voglmayr 2014	Saprotrophic
Tricholoma atrosquamosum Sacc.	Symbiotic
Tricholoma bonii Basso & Candusso	Symbiotic
Tricholoma cingulatum (Almfelt ex Fr.) Jacobashch, 1892	Symbiotic
Tricholoma equestre (L.) P.Kumm.	Symbiotic
Tricholoma focale (Fr.) Ricken	Symbiotic
Tricholoma matsutake (S.Ito & S.Imai) Singer	Symbiotic
Tricholoma matsutake (S.Ito & S.Imai) Singer	Symbiotic
Tricholoma pessundatum (Fr.) Quél.	Symbiotic
Tricholoma saponaceum (Fr.) P.Kumm.	Symbiotic
Tricholoma subacutum Peck 1889,	Symbiotic
Tricholoma transmutans (Peck) Sacc. 1887	Symbiotic
Tricholomopsis decora (Fr.) Singer 1939	Saprotrophic
Tricholomopsis flammula Métrod ex Holec, 2009	Saprotrophic
Tricholomopsis rutilans (Schaeff.) Singer, 1939	Saprotrophic
Trichophaea abundans (P.Karst.) Boud.	Saprotrophic
Truncocolumella rubra Zeller	Symbiotic
Tubaria romagnesiana Arnolds 1982	Saprotrophic
Tuber alboumbilicum Y.Wang & Shu H.Li	Symbiotic
Tuber liui A-S.Xu	Symbiotic
Tuber pacificum Trappe, Castellano & Bushnell	Symbiotic
Tuber wenchuanense L.Fan & J.Z.Cao	Symbiotic
Tuber yigongense L.Fan & W.P.Xiong	Symbiotic
Tulostoma simulans Lloyd	Saprotrophic
Typhula variabilis Riess	Saprotrophic
Tyromyces chioneus (Fr.) P.Karst.	Saprotrophic
Tyromyces kmetii (Bres.) Bondartsev & Singer 1941	Saprotrophic
Wynnella silvicola Nannf.	Saprotrophic
Xanthoporia radiata (Sowerby) Ţura, Zmitr., Wasser, Raats & Nevo	Saprotrophic
Xerocomellus chrysenteron (Bull.) Šutara	Symbiotic

Xerocomus ferrugineus (Schaeff.) Alessio	Symbiotic
Xerocomus longistipitatus K.Das, A.Parihar, D.Chakr. & Baghela	Symbiotic
Xerocomus nigromaculatus Hongo	Symbiotic
Xerocomus perplexus (Watling) Neves, Binder, Halling, Hibbett & Soytong 2012,	Symbiotic
Xeromphalina campanella (Batsch) Kühner & Maire	Saprotrophic
Xeromphalina tenuipes (Schwein.) A.H. Sm	Saprotrophic
Xylaria furcata Fr.	Saprotrophic
Xylaria grammica Mont.	Saprotrophic
Xylaria hypoxylon (L.) Grev.	Saprotrophic
Xylaria karsticola J.Fourn. & M.Stadler	Saprotrophic
Zangia olivaceobrunnea Yan C. Li& Zhu L. Yang	Symbiotic

Table S6. Toxic Fungal Species in the Nyang River Basin

**Scientific name**	**Poisoning type**
Bulgaria inquinans (Pers.) Fr.	Porphyrin toxins, photosensitive dermatitis type
Agaricus xanthodermus Genev.	Gastroenteritis
Lepiota castanea var. vinosobrunnea J.Aug.Schmitt	Liver and kidney damage
Lepiota cristata (Bolton) P.Kumm.	Unknown
Amanita flavipes S.Imai	Psychoneurological disorder
Amanita subfrostiana Zhu L.Yang	Psychoneurological disorder
Amanita vaginata (Bull.) Lam.	Unknown
Amanita orsonii Ash.Kumar & T.N.Lakh.	Psychoneurological disorder
Cortinarius gentilis (Fr.) Fr.	Renal failure type
Gliophorus irrigatus (Pers.) A.M.Ainsw. & P.M.Kirk	Unknown
Gliophorus psittacinus (Schaeff.) Herink	Psychoneurological disorder
Hygrocybe conica (Schaeff.) P.Kumm.	Gastroenteritis, psychoneurological disorder
Hygrocybe flavescens (Kauffman) Singer	Unknown
Gymnopilus aeruginosus (Peck) Singer	Gastroenteritis, psychoneurological disorder
Inocybe flavella P.Karst.	Unknown
Inocybe cincinnata (Fr.) Quél.	Psychoneurological disorder
Inocybe geophylla P.Kumm.	Psychoneurological disorder
Inocybe glabripes Ricken	Psychoneurological disorder
Inocybe lanuginosa (Bull.) P.Kumm.	Psychoneurological disorder
Inocybe pseudoteraturgus Vauras & Kokkonen	Psychoneurological disorder
Inocybe occulta Esteve-Rav., Bandini, B.Oertel & G.Moreno	Psychoneurological disorder
Inocybe plurabellae Bandini, B.Oertel & U.Eberh.	Psychoneurological disorder
Inocybe pseudorubens Carteret & Reumaux	Psychoneurological disorder
Inocybe sublilacina Matheny & A.Voitk	Unknown
Inosperma rosellicaulare (Grund & D.E.Stuntz) Matheny & Esteve-Rav.	Psychoneurological disorder

Mallocybe siciliana (Brugaletta, Consiglio & M.Marchetti) Brugaletta, Consiglio & M.Marchetti	Psychoneurological disorder
Mallocybe malenconii (R.Heim) Matheny & Esteve-Rav.	Psychoneurological disorder
Pseudosperma rimosum (Bull.) Matheny & Esteve-Rav.	Psychoneurological disorder, respiratory and circulatory failure
Mycena pura (Pers.) P.Kumm.	Gastroenteritis, psychoneurological disorder
Coprinellus micaceus (Bull.) Vilgalys, Hopple & Jacq.Johnson	Gastroenteritis, psychoneurological disorder
Coprinopsis atramentaria (Bull.) Redhead, Vilgalys & Moncalvo	Gastroenteritis
Coprinopsis lagopus (Fr.) Redhead, Vilgalys & Moncalvo	Unknown
Lacrymaria lacrymabunda (Bull.) Pat.	Gastroenteritis
Galerina fibrillosa A.H.Sm.	Liver and kidney damage, gastroenteritis, psychoneurological disorder, hemolysis
Hypholoma capnoides (Fr.) P.Kumm.	Unknown
Hypholoma fasciculare (Huds.) P.Kumm.	Liver and kidney damage, respiratory and circulatory failure, gastroenteritis
Pholiota lubrica (Pers.) Singer	Gastroenteritis
Pholiota squarrosa (Vahl) P.Kumm.	Gastroenteritis, psychoneurological disorder
Pholiota multicingulata E.Horak	Gastroenteritis
Tricholoma equestre (L.) P.Kumm.	Rhabdomyolysis type
Tricholoma pessundatum (Fr.) Quél.	Gastroenteritis
Tricholoma saponaceum (Fr.) P.Kumm.	saponaceolode A
Exidia glandulosa (Bull.) Fr.	Gastroenteritis
Caloboletus panniformis (Taneyama & Har.Takah.) Vizzini	Gastroenteritis
Gyroporus castaneus (Bull.) Quél.	Unknown
Paxillus involutus (Batsch) Fr.	Hemolysis
Suillus viscidus (L.) Roussel	Gastroenteritis
Suillus luteus (L.) Roussel	Gastroenteritis, hemolysis
Tapinella panuoides (Fr.) E.-J.Gilbert	Gastroenteritis
Lactarius hirtipes J.Z.Ying	Gastroenteritis
Lactarius torminosus (Schaeff.) Pers.	Gastroenteritis
Lactarius pubescens Fr.	Gastroenteritis
Lactarius alpinihirtipes X.H.Wang	Gastroenteritis
Lactarius purpureus R.Heim	Gastroenteritis
Lactarius aquizonatus Kytöv.	Unknown
Lactifluus pilosus (Verbeken, H.T.Le & Lumyong) Verbeken	Gastroenteritis
Russula queletii f. albocitrina Barbier	Gastroenteritis
Agaricus tibetensis J.L. Zhou & R.L. Zhao 2016,	Gastroenteritis
Amanita fritillaria(Berk.)Sacc.	Unknown

Amanita muscaria (L.:Fr.) Pers.ex Hook.	Psychoneurological disorder
Amanita subglobosa Zhu L. Yang 1997	Psychoneurological disorder
Amanita subjunquillea S.Imail	Acute liver injury type
Candolleomyces candolleanus (Fr.) D. Wächt. & A. Melzer 2020,	Psychoneurological disorder
Collybia phyllophila (Pers.) Z.M. He & Zhu L. Yang, 2023 in [He Z et al. (2023)]	Psychoneurological disorder
Coprinopsis nivea (Pers.) Redhead et.al	Unknown
Coprinus comatus var. caprimammillatus	Unknown
Cortinarius castaneus (Bull.) Fr.	Unknown
Cortinarius cinnamomeus (L.) Fr.	Unknown
Cortinarius sanguineus (Wulfen.) Fr.	Renal failure type
Galerina fasciculata Hongo	Acute liver injury type
Gomphus orientalis R.H. Petersen & M. Zang	Gastroenteritis
Gymnopus dryophilus JGI SC001DCMIDF	Gastroenteritis
Gyromitra infula (Schaeff.et Fr.) Qul.	Gastroenteritis, psychoneurological disorder,acute hepatorenal injury
Helvella elastica Bull.	Liver and kidney damage, gastroenteritis
Infundibulicybe gibba (Pers.) P. Kumm. 1871	Gastroenteritis, psychoneurological disorder,respiratory and circulatory failure,
Inocybe flocculosa var. flocculosa	Unknown
Inocybe lacera var. lacera	Psychoneurological disorder
Inocybe nitidiuscula (Britzelm.) Lapl., 1894	Psychoneurological disorder
Inocybe splendentoides Bon 1990	Unknown
Ionomidotis fulvotingens (Berk. & M.A. Curtis) E.K. Cash 1939,	Gastroenteritis；Photosensitive dermatitis type
Lactarius rufus (Scop.) Fr.	Gastroenteritis
Lactarius scrobiculatus var. pubescens	Gastroenteritis
Leccinum scabrum (Bull.et Fr.) Gray	Gastroenteritis
Legaliana badia (Pers.) Van Vooren 2020	Liver and kidney damage, gastroenteritis, psychoneurological disorder, hemolysis
Otidea cochleata (L.) Fuckel	Unknown
Panellus stipticus (Bull.) P. Karst. 1879	Gastroenteritis
Phaeolepiota aurea (Bull.) R. Maire ex Konrad & Maubl. 1928	Gastroenteritis
Pholiota alnicola (Fr.) Singer	Unknown
Protostropharia semiglobata (Batsch) Redhead, Moncalvo & Vilgalys, 2013	Psychoneurological disorder
Psilocybe coprophila (Bull.et Fr.) Kummer	Psychoneurological disorder
Russula emetica (Schaeff.) Pers. 1796	Gastroenteritis
Russula farinipes Romell.	Unknown
Russula foetens Pers., 1796	Gastroenteritis
Russula lutea (Huds.) Gray 1821	Unknown
Russula subnigricans Hongo	Rhabdomyolysis type

Scleroderma areolatum Ehrenb. 1818	Gastroenteritis, psychoneurological disorder
Tricholomopsis rutilans (Schaeff.) Singer, 1939	Gastroenteritis

Table S7. Statistics on the Threat Levels of Large Fungi in the Nyang River Basin

**Scientific name**	**Protection level**
Gyromitra infula (Schaeff.et Fr.) Qul.	LC
Helvella elastica Bull.	LC
Peziza succosa Berk. 1841	DD
Scutellinia scutellata (L.) Lambotte	LC
Otidea cochleata (L.) Fuckel	LC
Otidea olivaceobrunnea Harmaja 2009,	DD
Tarzetta catinus (Holmsk.) Korf & J.K. Rogers 1971	LC
Pseudoplectania nigrella (Pers.) Fuckel	LC
Ionomidotis fulvotingens (Berk. & M.A. Curtis) E.K. Cash 1939,	DD
Mitrula brevispora Zheng Wang	DD
Cudonia circinans (Pers.) Fr. 1849	LC
Cudonia lutea (Peck) Sacc. 1885	LC
Spathularia flavida Persoon, 1797	LC
Cordyceps cylindrica Petch	DD
Xylaria furcata Fr.	LC
Xylaria grammica Mont.	LC
Xylaria hypoxylon (L.) Grev.	LC
Agaricus abruptibulbus Peck, 1905	LC
Agaricus arvensis Schaeff., 1774	DD
Agaricus bisporus (J.E. Lange) Imbach	DD
Agaricus campestris L. :Fr.	LC
Agaricus comtulus Fr., 1838	LC
Agaricus sylvaticus var. occidentalis (Kerrigan) Blanco-Dios,	LC
Coprinopsis nivea (Pers.) Redhead et.al	LC
Coprinus comatus var. caprimammillatus	LC
Crucibulum laeve (Huds.) Kambly, 1936	LC
Cyathus lijiangensis T.X. Zhou & R.L. Zhao 2004	DD
Cyathus striatus (Huds.) Willd.	LC
Leucoagaricus nympharum (Kalchbr.) Bon	LC
Lycoperdon molle Pers., 1801	DD
Lycoperdon pratense Pers.	DD
Amanita atrofusca Zhu L. Yang	LC
Amanita flavoconia G. F. Atk., 1902	DD
Amanita fritillaria(Berk.)Sacc.	LC
Amanita muscaria (L.:Fr.) Pers.ex Hook.	LC

Amanita orientifulva Zhu L. Yang, M. Weiss & Oberw. 2004	LC
Amanita subglobosa Zhu L. Yang 1997	LC
Limacella ochraceolutea P.D. Orton	LC
Auriscalpium vulgare Gray	LC
Clavulinopsis fusiformis (Sowerby) Corner	LC
Cortinarius alboviolaceus (Pers.) Fr.	DD
Cortinarius anomalus Fr.	DD
Cortinarius canabarba Moser	LC
Cortinarius castaneus (Bull.) Fr.	DD
Cortinarius cinnamomeus (L.) Fr.	LC
Cortinarius citrinoolivaceus Mos.	DD
Cortinarius croceus (Schaeff.) Gray 1821	DD
Cortinarius gentilis (Fr.) Fr. 1838	DD
Cortinarius glaucopus sensu Rea (1922)	DD
Cortinarius longipes Peck	DD
Cortinarius multiformis sensu NCL (1960)	DD
Cortinarius pholideus (Lilj.) Fr. 1838,	LC
Cortinarius purpurascens Fr.	DD
Cortinarius rapaceus Fr.	DD
Cortinarius sanguineus (Wulfen.) Fr.	DD
Cortinarius similis (E. Horak) Peintner, E. Horak, M.M. Moser & Vilgalys 2002	DD
Cortinarius torvus (Fr.) Fr., 1838	DD
Cortinarius violaceus (L.) Fr.	LC
Galerina clavata (Velen.) KA%BChner 1935	DD
Galerina fasciculata Hongo	LC
Hebeloma vaccinum Romagn., 1965	DD
Leucocortinarius bulbiger (Alb. & Schwein.) Singer 1945	LC
Laccaria laccata (Scop. Et Fr.)	LC
Laccaria proxima (Boud.) Pat.	LC
Hygrophorus imazeki (Hongo) Hongo	DD
Panaeolus fimicola (Pers.) Gillet	LC
Crepidotus mollis (Schaeff.) Staude	LC
Inocybe abjecta Sacc., 1887	DD
Inocybe adaequata (Britzelm.) Sacc.	LC
Inocybe curvipes P. Karst.	LC
Inocybe flocculosa var. flocculosa	DD
Inocybe lacera var. lacera	LC
Inocybe montana Kobayasi	DD
Inocybe nitidiuscula (Britzelm.) Lapl., 1894	LC
Campanella tristis (G. Stev.) Segedin 1993	LC
Marasmius maximus Hongo	LC
Mycena haematopus (Pers.) P. Kumm. 1871	LC
Mycena laevigata (Lasch) Gillet 1876	DD
Mycena leaiana sensu auct. NZ	DD

Mycena sanguinolenta (Alb. & Schwein.) P. Kumm., 1871	LC
Mycena viridimarginata P. Karst.	DD
Panellus stipticus (Bull.) P. Karst. 1879	LC
Roridomyces roridus (Fr.) Rexer 1994	LC
Xeromphalina campanella (Batsch) Kühner & Maire	LC
Xeromphalina tenuipes (Schwein.) A.H. Sm	LC
Gymnopus aquosus (Bull.) Antonín & Noordel. 1997	LC
Gymnopus dryophilus JGI SC001DCMIDF	LC
Rhodocollybia butyracea (Bull.) Lennox	LC
Armillaria borealis Marxm. & Korhonen 1982,	LC
Armillaria cepistipes Velen., 1920	DD
Armillaria gallica Marxm. & Romagn., 1987	DD
Strobilurus occidentalis V.L. Wells & Kempton 1971,	DD
Hohenbuehelia grisea (Peck) Singer 1951	DD
Hohenbuehelia subreniformis (Thorn & G.L. Barron) Thorn 2013	DD
Phyllotopsis nidulans (Pers.) Singer 1936	LC
Pleurotus calyptratus (Lindblad.) Sacc.	LC
Pleurotus cornucopiae (Pual. Et Pers.) Roll.	LC
Pleurotus spodoleucus Fr.	LC
Pluteus pouzarianus Singer	DD
Coprinellus xanthothrix (Romagn.) Vilgalys, Hopple & Jacq. Johnson 2001,	DD
Psathyrella bipellis (Quelet) A.H. Smith	DD
Psathyrella candolleana (Fr.) Maire, 1937	LC
Psathyrella pygmaea (Bull.) Singer	DD
Schizophyllum commune 4.8 streak	LC
Agrocybe pediades f. bispora	LC
Pholiota alnicola (Fr.) Singer	LC
Protostropharia semiglobata (Batsch) Redhead, Moncalvo & Vilgalys, 2013	LC
Arrhenia epichysium (Pers.) Redhead, Lutzoni, Moncalvo & Vilgalys 2002	LC
Arrhenia spathulata (Fr.) Redhead	DD
Clitocybe diatreta (Fr.) P. Kumm., 1871	DD
Clitocybe vibecina (Fr.) Quel. 1872	DD
Melanoleuca brevipes (Bull.) Pat	LC
Melanoleuca verrucipes (Fr.) Singer 1939	LC
Phaeolepiota aurea (Bull.) R. Maire ex Konrad & Maubl. 1928	LC
Tricholoma cingulatum (Almfelt ex Fr.) Jacobashch, 1892	LC
Tricholomopsis decora (Fr.) Singer 1939	LC
Tricholomopsis rutilans (Schaeff.) Singer, 1939	LC
Pistillaria petasitis S. Imai	DD
Chalciporus piperatus (Bull.) Bataille 1908	DD
Leccinum rugosiceps (Peck) Singer 1945,	LC
Leccinum scabrum (Bull.et Fr.) Gray	LC
Xerocomellus chrysenteron (Bull.) Šutara	DD
Zangia olivaceobrunnea Yan C. Li& Zhu L. Yang	DD

Gyroporus longicystidiatus Nagas. & Hongo	LC
Gomphidius glutinosus (Schaeff.) Fr. 1838	LC
Scleroderma areolatum Ehrenb. 1818	LC
Cantharellus cibarius Fr.	DD
Craterellus cornucopioides var. mediosporus	LC
Gomphus orientalis R.H. Petersen & M. Zang	NT
Sarcodon scabrosus(Fr.) P. Karst.	LC
Geastrum velutinum Morgan	LC
Gloeophyllum sepiarium (Wulfen) P. Karst. 1882	LC
Gloeophyllum striatum (Swartz) Murrill	LC
Ramaria distinctissima R.H. Petersen & M. Zang	NT
Ramaria hemirubella Petersen et Zang	NT
Hymenochaete cruenta (Pers.)Donk	LC
Hymenochaete sphaericola Lloyd	DD
Postia hirsuta L.L. Shen & B.K. Cui 2014	DD
Ganoderma applanatum (Pers.) Pat.	NT
Bjerkandera adusta (Willd.,) P.Karst.	LC
Gloeoporus taxicola (Pers.)Gilb.& Ryvarden	LC
Irpex lacteus (Fr.) Fr., 1828	LC
Daedaleopsis confragosa	LC
Daedaleopsis tricolor (Bull.) Bondartsev &Singer	LC
Lentinus sajor-caju Fr.	LC
Neofavolus alveolaris (DC.) Sotome & T. Hatt. 2012	LC
Panus conchatus （Bull.）Fr.	LC
Perenniporia fraxinea (Bull.) Ryvarden	LC
Perenniporia tibetica B.K. Cui&C.L. Zhao	DD
Polyporus arcularius （Batsch）Fr.	LC
Polyporus tuberaster (Jacq. ex Pers.) Fr. 1821	LC
Polyporus umbellatus (Pers.) Fr.	LC
Fuscoporia setifer (T. Hatt.) Y.C.Dai	DD
Pycnoporus cinnabarinus (Jacq.) P.Karst.	LC
Pycnoporus sanguineus (L.) Murrill	LC
Trametes gibbosa (Pers.) Fr.	LC
Trametes ochracea (Pers.) Gilb.& Ryvarden	LC
Trametes versicolor FP-101664 SS1	LC
Tyromyces kmetii (Bres.) Bondartsev & Singer 1941	DD
Sparassis crispa (Wulfen) Fr. 1819	LC
Lentinellus flabelliformis (Bolton) S. Ito 1959	LC
Heterobasidion insulare (Murrill) Ryvarden 1972	LC
Heterobasidion orientale Tokuda, T. Hatt. & Y.C. Dai 2009,	LC
Lactarius hysginus Fr.	LC
Lactarius indigo (Schw.) Fr.	LC
Lactarius piperatus (Scop.) Fr.	LC
Lactarius pterosporus Romagn., 1949	DD

Lactarius rufus (Scop.) Fr.	LC
Lactarius scrobiculatus var. pubescens	LC
Lactarius trivialis (Fr.) Fr. 1838	DD
Russula azurea Bres. 1882	LC
Russula chiui G.J. Li & H.A. Wen 2015	DD
Russula emetica (Schaeff.) Pers. 1796	LC
Russula farinipes Romell.	LC
Russula foetens Pers., 1796	LC
Russula olivacea Pers., 1796	LC
Russula paludosa Britzelm.	LC
Russula rubra (Krombh.) Bres.	LC
Russula subnigricans Hongo	LC
Russula xerampelina var. xerampelina	LC
Aleurodiscus wakefieldiae Boidin & Beller 1967	DD
Stereum ochraceoflavum (Schwein.) Sacc., 1888	DD
Stereum sanguinolentum (Alb. & Schwein.) Fr. 1838	DD
Auricularia fuscosuccinea (Mont.) Henn., 1893	LC
Dacryopinax spathularia (Schwein.) G.W. Martin, 1948	LC
Guepiniopsis buccina (Pers.) L.L. Kenn. 1959,	LC
Dacrymyces australis Lloyd 1920	DD
Naematelia aurantialba (Bandoni & M. Zang) Millanes & Wedin	VU
Agaricus benesii (Pilát) Pilát	LC
Agaricus subrutilescens (Kauffman) Hotson & D.E.Stuntz	LC
Agaricus xanthodermus Genev.	LC
Aleuria aurantia (Pers.) Fuckel	LC
Amanita battarrae (Boud.) Bon	LC
Amanita brunneofuliginea Zhu L.Yang	LC
Amanita citrinoindusiata Zhu L.Yang, Y.Y.Cui & Q.Cai	DD
Amanita crocea (Quél.) Singer	DD
Amanita flavipes S.Imai	LC
Amanita nivalis Grev.	LC
Amanita orsonii Ash.Kumar & T.N.Lakh.	LC
Amanita subfrostiana Zhu L.Yang	DD
Amanita vaginata (Bull.) Lam.	LC
Amyloporia sinuosa (Fr.) Rajchenb., Gorjón & Pildain	LC
Arrhenia acerosa (Fr.) Kühner	LC
Astraeus koreanus (V.J.Staněk) Kreisel	LC
Atheniella adonis (Bull.) Redhead, Moncalvo, Vilgalys, Desjardin & B.A.Perry	LC
Boletus reticulatus Schaeff.	DD
Boletus reticuloceps (M.Zang, M.S.Yuan & M.Q.Gong) Q.B.Wang & Y.J.Yao	LC
Bondarzewia mesenterica (Schaeff.) Kreisel	LC
Bovista plumbea Pers.	LC
Bulgaria inquinans (Pers.) Fr.	LC
Caloboletus panniformis (Taneyama & Har.Takah.) Vizzini	DD

Calvatia gigantea (Batsch) Lloyd	LC
Ceraceomyces serpens (Tode) Ginns	DD
Cerrena unicolor (Bull.) Murrill	LC
Chamonixia caespitosa Rolland	DD
Cheilymenia theleboloides (Alb. & Schwein.) Boud.	LC
Chroogomphus confusus Yan C.Li & Zhu L.Yang	NT
Cinereomyces lindbladii (Berk.) Jülich	LC
Clavaria acuta Sowerby	DD
Clavaria californica R.H.Petersen	DD
Clavaria citrinorubra R.H.Petersen	DD
Clavaria flavipes Pers.	LC
Clavaria fragilis Holmsk.	LC
Clavaria fumosa Pers.	DD
Clavaria tenuipes Berk. & Broome	LC
Clavaria zollingeri Lév.	LC
Clavulina amethystina (Bull.) Donk	DD
Clavulina castaneipes (G.F.Atk.) Corner	DD
Clavulina coralloides (L.) J.Schröt.	LC
Clavulina rugosa (Bull.) J.Schröt.	LC
Clavulinopsis corniculata (Schaeff.) Corner	LC
Clavulinopsis helvola (Pers.) Corner	LC
Clavulinopsis laeticolor (Berk. & M.A.Curtis) R.H.Petersen	DD
Clavulinopsis luteoalba (Rea) Corner	DD
Clitocybe metachroa (Fr.) P.Kumm.	DD
Clitopilus hobsonii (Berk.) P.D.Orton	DD
Clitopilus passeckerianus (Pilát) Singer	DD
Clitopilus prunulus (Scop.) P.Kumm.	LC
Clitopilus scyphoides (Fr.) Singer	DD
Coniophora fusispora (Cooke & Ellis) Cooke	DD
Conocybe dumetorum (Velen.) Svrček	DD
Conocybe pilosella (Pers.) Kühner	DD
Coprinellus disseminatus (Pers.) J.E.Lange	LC
Coprinellus micaceus (Bull.) Vilgalys, Hopple & Jacq.Johnson	LC
Coprinellus radians (Desm.) Vilgalys, Hopple & Jacq.Johnson	LC
Coprinopsis atramentaria (Bull.) Redhead, Vilgalys & Moncalvo	LC
Coprinopsis lagopides (P.Karst.) Redhead, Vilgalys & Moncalvo	DD
Coprinopsis lagopus (Fr.) Redhead, Vilgalys & Moncalvo	LC
Coprinopsis stercorea (Fr.) Redhead, Vilgalys & Moncalvo	DD
Cortinarius anomalus (Fr.) Fr.	DD
Cortinarius anthracinus (Fr.) E.Berger	DD
Cortinarius armillatus (Fr.) Fr.	DD
Cortinarius balaustinus Fr.	DD
Cortinarius betuletorum M.M.Moser	DD
Cortinarius caninus (Fr.) Fr.	DD

Cortinarius claricolor (Fr.) Fr.	DD
Cortinarius gentilis (Fr.) Fr.	DD
Cortinarius obtusus (Fr.) Fr.	DD
Crepidotus applanatus (Pers.) P.Kumm.	LC
Crepidotus crocophyllus (Berk.) Sacc.	DD
Crepidotus malachius Sacc.	DD
Cuphophyllus pratensis (Pers.) Bon	LC
Cuphophyllus virgineus (Wulfen) Kovalenko	LC
Cystolepiota sistrata (Fr.) Singer ex Bon & Bellù	DD
Diplomitoporus flavescens (Bres.) Domański	DD
Entoloma alboumbonatum Hesler	DD
Entoloma byssisedum (Pers.) Donk	LC
Entoloma conferendum (Britzelm.) Noordel.	LC
Entoloma longistriatum (Peck) Noordel.	DD
Entoloma neglectum (Lasch) Arnolds	DD
Entoloma politum (Pers.) Noordel.	DD
Entoloma prunuloides (Fr.) Quél.	DD
Entoloma pulchellum (Hongo) Hongo	DD
Entoloma sericeum f. flexipes (J.Favre) E.Horak	DD
Entoloma serrulatum (Fr.) Hesler	DD
Entoloma undatum (Gillet) M.M.Moser	DD
Exidia glandulosa (Bull.) Fr.	LC
Exidia saccharina Fr.	LC
Exidia thuretiana (Lév.) Fr.	DD
Exidiopsis calcea (Pers.) K.Wells	DD
Exidiopsis effusa (Bref. ex Sacc.) Möller	LC
Fayodia bisphaerigera (J.E.Lange) Singer	DD
Galerina fibrillosa A.H.Sm.	DD
Galiella amurensis (Lj.N.Vassiljeva) Raitv.	LC
Ganoderma australe (Fr.) Pat.	LC
Ganoderma leucocontextum T.H.Li, W.Q.Deng, Sheng H.Wu, Dong M.Wang & H.P.Hu	LC
Gautieria globispora K.Tao, Ming C.Chang & B.Liu	DD
Gautieria morchelliformis Vittad.	DD
Geoglossum fallax var. subpumilum (S.Imai) S.Imai	LC
Geoglossum glabrum f. sphagnophilum (Ehrenb.) J.Favre	DD
Geoglossum umbratile var. heterosporum (Mains) Maas Geest.	LC
Geopora tenuis (Fuckel) T.Schumach.	DD
Gliophorus irrigatus (Pers.) A.M.Ainsw. & P.M.Kirk	DD
Gliophorus psittacinus (Schaeff.) Herink	LC
Gymnopilus aeruginosus (Peck) Singer	LC
Gymnopilus bellulus (Peck) Murrill	DD
Gymnopilus luteofolius (Peck) Singer	DD
Gymnopilus sapineus (Fr.) Murrill	LC

Gyroporus castaneus (Bull.) Quél.	LC
Haploporus odorus (Sommerf.) Bondartsev & Singer	DD
Hebeloma sordescens Vesterh.	DD
Hebeloma testaceum (Fr.) Quèl.	DD
Helvella lacunosa Afzel.	LC
Helvella maculata N.S.Weber	LC
Helvella philonotis Dissing	DD
Helvellosebacina concrescens (Schwein.) Oberw., Garnica & K.Riess	DD
Hemimycena angustispora (P.D.Orton) Singer	DD
Hemimycena gracilis (Quél.) Singer	DD
Hemimycena ochrogaleata (J.Favre) M.M.Moser	DD
Henningsomyces candidus (Pers.) Kuntze	DD
Hohenbuehelia longipes (Boud.) M.M.Moser	DD
Hortiboletus rubellus (Krombh.) Simonini, Vizzini & Gelardi 2015	LC
Humaria hemisphaerica (F.H.Wigg.) Fuckel	LC
Hydnellum caeruleum (Hornem.) P.Karst.	DD
Hydnellum concrescens (Pers.) Banker	LC
Hydnellum spongiosipes (Peck) Pouzar	DD
Hydnotrya cerebriformis Harkn.	LC
Hydropus moserianus Bas	DD
Hygrocybe acutoconica (Clem.) Singer	LC
Hygrocybe cantharellus (Schwein.) Murrill	LC
Hygrocybe ceracea (Sowerby) P.Kumm.	LC
Hygrocybe coccineocrenata (P.D.Orton) M.M.Moser	DD
Hygrocybe conica (Schaeff.) P.Kumm.	LC
Hygrocybe flavescens (Kauffman) Singer	LC
Hygrocybe insipida (J.E.Lange) M.M.Moser	DD
Hygrocybe miniata (Fr.) P.Kumm.	LC
Hygrocybe nigrescens (Quél.) Kühner	LC
Hygrophorus discoideus (Pers.) Fr.	DD
Hygrophorus olivaceoalbus (Fr.) Fr.	LC
Hygrophorus persicolor Ricek	DD
Hygrophorus purpurascens (Alb. & Schwein.) Fr.	DD
Hygrophorus pustulatoides Lebeuf, E.Larss. & Bellanger	DD
Hymenochaete tenuis Peck	LC
Hyphodontia pallidula (Bres.) J.Erikss.	DD
Hypholoma capnoides (Fr.) P.Kumm.	LC
Hypholoma fasciculare (Huds.) P.Kumm.	LC
Hypoxylon rutilum Tul. & C.Tul.	LC
Inocybe calospora Quél.	LC
Inocybe cincinnata (Fr.) Quél.	DD
Inocybe dulcamara (Pers.) P.Kumm.	DD
Inocybe flavella P.Karst.	DD
Inocybe geophylla P.Kumm.	LC

Inocybe glabripes Ricken	DD
Inocybe grammatoides Esteve-Rav., Pancorbo & E.Rubio	LC
Inocybe involuta Kuyper	DD
Inocybe ionochlora Romagn.	DD
Inocybe lanuginosa (Bull.) P.Kumm.	LC
Inocybe lutescens Velen.	DD
Inocybe mixtilis (Britzelm.) Sacc.	DD
Inocybe muricellata Bres.	DD
Inocybe obscurobadia (J.Favre) Grund & D.E.Stuntz	DD
Inocybe ochroalba Bruyl.	DD
Inocybe petiginosa (Fr.) Gillet	DD
Inocybe rimosa (Bull.) P.Kumm.	LC
Inocybe rufoalba Sacc.	DD
Inocybe vulpinella Bruyl.	DD
Inocybe xanthomelas Boursier & Kühner	LC
Jaapia ochroleuca (Bres.) Nannf. & J.Erikss.	LC
Kavinia alboviridis (Morgan) Gilb. & Budington	DD
Kuehneromyces mutabilis (Schaeff.) Singer & A.H.Sm.	LC
Laccaria acanthospora A.W.Wilson & G.M.Muell.	DD
Laccaria alba Zhu L.Yang & Lan Wang	NT
Laccaria bicolor (Maire) P.D.Orton	LC
Laccaria fulvogrisea Popa, Rexer & G.Kost	DD
Laccaria negrimarginata A.W.Wilson & G.M.Muell.	DD
Laccaria pumila Fayod	DD
Laccaria salmonicolor A.W.Wilson & G.M.Muell.	DD
Laccaria tortilis (Bolton) Cooke	LC
Lacrymaria lacrymabunda (Bull.) Pat.	LC
Lactarius aurantiacus (Pers.) Gray	LC
Lactarius badiosanguineus Kühner & Romagn.	DD
Lactarius deterrimus Gröger	LC
Lactarius glyciosmus (Fr.) Fr.	LC
Lactarius hirtipes J.Z.Ying	DD
Lactarius picinus Fr.	LC
Lactarius pubescens Fr.	LC
Lactarius spinosulus Quél. & Le Bret.	DD
Lactarius torminosus (Schaeff.) Pers.	LC
Lentinellus cochleatus (Pers.) P.Karst.	LC
Lentinellus ursinus (Fr.) Kühner	LC
Lentinus arcularius (Batsch) Zmitr.	DD
Leotia lubrica (Scop.) Pers.	LC
Lepiota castanea var. vinosobrunnea J.Aug.Schmitt	LC
Lepiota coloratipes Vizzini, J.F.Liang, Jančovič. & Zhu L.Yang	DD
Lepiota cortinarius J.E.Lange	LC
Lepiota cristata (Bolton) P.Kumm.	LC

Lepiota rufipes f. phaeophylla Bon	DD
Lepista nuda (Bull.) Cooke	LC
Lepista panaeolus (Fr.) P.Karst.	DD
Lepista sordida (Schumach.) Singer	LC
Leucophleps spinispora Fogel	DD
Lycoperdon dermoxanthum Vittad.	DD
Lycoperdon excipuliforme (Scop.) Pers.	LC
Lycoperdon mammiforme Pers.	LC
Lycoperdon perlatum Pers.	LC
Lycoperdon pratense Pers.	DD
Lycoperdon pyriforme Schaeff.	DD
Lycoperdon subumbrinum Jeppson & E.Larss.	LC
Macrocystidia cucumis (Pers.) Joss.	LC
Mallocybe terrigena (Fr.) Matheny, Vizzini & Esteve-Rav.	LC
Melanophyllum haematospermum (Bull.) Kreisel	DD
Mycena abramsii (Murrill) Murrill	LC
Mycena acicula (Schaeff.) P.Kumm.	LC
Mycena amicta (Fr.) Quél.	DD
Mycena metata (Fr.) P.Kumm.	DD
Mycena pura (Pers.) P.Kumm.	LC
Mycena rosella (Fr.) P.Kumm.	DD
Mycena stylobates (Pers.) P.Kumm.	LC
Mycenella bryophila (Voglino) Singer	DD
Mycenella lasiosperma (Bres.) Locq.	DD
Mycetinis scorodonius (Fr.) A.W.Wilson & Desjardin	LC
Naucoria bohemica Velen.	DD
Otidea alutacea (Pers.) Massee	LC
Otidea bufonia (Pers.) Boud.	LC
Panaeolus alcis M.M.Moser	LC
Parasola plicatilis (Curtis) Redhead, Vilgalys & Hopple	LC
Paxillus involutus (Batsch) Fr.	LC
Peniophora cinerea (Pers.) Cooke	DD
Peniophora incarnata (Pers.) P.Karst.	LC
Peziza badia var. terrestris Alb. & Schwein.	LC
Peziza depressa var. applanata (Hedw.) Pers.	DD
Peziza fimeti (Fuckel) E.C.Hansen	DD
Peziza howsei Boud. ex Donadini	DD
Peziza limnaea Maas Geest.	DD
Peziza michelii (Boud.) Dennis	DD
Peziza nivalis (R.Heim & L.Rémy) M.M.Moser	DD
Peziza ostracoderma Korf	DD
Peziza saniosa Schrad.	DD
Phallus impudicus L.	LC
Phellodon melaleucus (Sw. ex Fr.) P.Karst.	DD

Phellodon niger (Fr.) P.Karst.	DD
Phlebia acerina Peck	DD
Phlebia tremellosa (Schrad.) Nakasone & Burds.	LC
Pholiota gummosa (Lasch) Singer	LC
Pholiota lenta (Pers.) Singer	LC
Pholiota lubrica (Pers.) Singer	LC
Pholiota spumosa (Fr.) Singer	LC
Pholiota squarrosa (Vahl) P.Kumm.	LC
Plectania melastoma (Sowerby) Fuckel	LC
Plectania rhytidia (Berk.) Nannf. & Korf	DD
Pluteus cervinus (Schaeff.) P.Kumm.	LC
Pluteus cinereofuscus J.E.Lange	DD
Pluteus semibulbosus (Lasch) Quél.	LC
Porodaedalea cancriformans (M.J.Larsen, Lombard & Aho) T.Wagner & M.Fisch.	DD
Postia balsamea (Peck) Jülich	LC
Postia caesia (Schrad.) P.Karst.	LC
Postia fragilis (Fr.) Jülich	LC
Postia ptychogaster (F.Ludw.) Vesterh.	DD
Postia rennyi (Berk. & Broome) Rajchenb.	DD
Postia sericeomollis (Romell) Jülich	LC
Postia subcaesia (A.David) Jülich	LC
Protoglossum niveum (Vittad.) T.W.May	DD
Psathyrella corrugis (Pers.) Konrad & Maubl.	LC
Pseudotomentella atrofusca M.J.Larsen	DD
Ramariopsis crocea (Pers.) Corner	LC
Ramariopsis kunzei (Fr.) Corner	LC
Rectipilus davidii (D.A.Reid) Agerer	DD
Resupinatus applicatus (Batsch) Gray	LC
Rhizopogon roseolus (Corda) Th.Fr.	LC
Russula acrifolia Romagn.	LC
Russula adusta (Pers.) Fr.	LC
Russula aeruginea Lindblad ex Fr.	LC
Russula atropurpurea (Krombh.) Britzelm.	LC
Russula azurea Bres.	LC
Russula brevipes Peck	LC
Russula brunneola Burl.	DD
Russula cessans A.Pearson	DD
Russula chloroides (Krombh.) Bres.	LC
Russula crustosa Peck, 1887	LC
Russula cuprea Krombh.	DD
Russula cyanoxantha (Schaeff.) Fr.	LC
Russula delica Fr.	LC
Russula exalbicans (Pers.) Melzer & Zvára	LC
Russula gracillima Jul.Schäff.	LC

Russula heterochroa Kühner	LC
Russula innocua (Singer) Romagn. ex Bon	DD
Russula nigricans Fr.	LC
Russula nitida (Pers.) Fr.	LC
Russula nobilis Velen.	LC
Russula odorata Romagn.	DD
Russula puellaris Fr.	LC
Russula punicea W.F.Chiu	DD
Russula queletii f. albocitrina Barbier	DD
Russula risigallina (Batsch) Sacc.	LC
Russula romellii Maire	LC
Russula sanguinea Fr.	LC
Russula turci var. gilva Einhell.	LC
Russula velenovskyi Melzer & Zvára	LC
Russula vesca Fr.	LC
Sarcodon imbricatus (L.) P.Karst.	LC
Sarcodon leucopus (Pers.) Maas Geest. & Nannf.	DD
Scleroderma bovista Fr.	LC
Sebacina candida L.S.Olive	DD
Sebacina epigaea (Berk. & Broome) Bourdot & Galzin	DD
Sebacina incrustans (Pers.) Tul. & C.Tul.	LC
Serpula himantioides (Fr.) P.Karst.	LC
Simocybe haustellaris (Fr.) Watling	DD
Sistotrema confluens Pers.	DD
Sistotrema muscicola (Pers.) S.Lundell	DD
Sowerbyella rhenana (Fuckel) J.Moravec	LC
Steccherinum laeticolor (Berk. & M.A.Curtis) Banker	DD
Steccherinum ochraceum (Pers. ex J.F.Gmel.) Gray	LC
Stereum hirsutum (Willd.) Pers.	LC
Suillus cavipes (Klotzsch) A.H.Sm. & Thiers	LC
Suillus luteus (L.) Roussel	LC
Suillus viscidus (L.) Roussel	LC
Tapinella panuoides (Fr.) E.-J.Gilbert	LC
Thelephora anthocephala (Bull.) Fr.	DD
Thelephora caryophyllea (Schaeff.) Pers.	LC
Thelephora palmata (Scop.) Fr.	LC
Thelephora terrestris Ehrh. ex Fr.	LC
Tolypocladium inegoense (Kobayasi) C.A.Quandt, Kepler & Spatafora	LC
Tolypocladium inflatum W.Gams	DD
Tolypocladium ophioglossoides (J.F.Gmel.) C.A.Quandt, Kepler & Spatafora	LC
Tomentella bryophila (Pers.) M.J.Larsen	DD
Tomentella coerulea Höhn. & Litsch.	DD
Tomentella ellisii (Sacc.) Jülich & Stalpers	DD
Tomentella ferruginea (Pers.) Pat.	DD

Tomentella lateritia Pat.	DD
Tomentella lilacinogrisea Wakef.	DD
Tomentella stuposa (Link) Stalpers	DD
Tomentella terrestris (Berk. & Broome) M.J.Larsen	DD
Trametes cubensis (Mont.) Sacc.	DD
Trametes trogii Berk.	LC
Trechispora cohaerens (Schwein.) Jülich & Stalpers	DD
Trechispora hymenocystis (Berk. & Broome) K.H.Larss.	DD
Trechispora microspora (P.Karst.) Liberta	DD
Trechispora mollusca (Pers.) Liberta	LC
Tremellodendropsis tuberosa (Grev.) D.A.Crawford	DD
Trichaptum abietinum (Pers. ex J.F.Gmel.) Ryvarden	LC
Tricholoma atrosquamosum Sacc.	DD
Tricholoma equestre (L.) P.Kumm.	LC
Tricholoma focale (Fr.) Ricken	DD
Tricholoma matsutake (S.Ito & S.Imai) Singer	VU
Tricholoma pessundatum (Fr.) Quél.	LC
Tricholoma saponaceum (Fr.) P.Kumm.	LC
Tuber alboumbilicum Y.Wang & Shu H.Li	NT
Tuber liui A-S.Xu	DD
Tuber wenchuanense L.Fan & J.Z.Cao	NT
Tulostoma simulans Lloyd	DD
Typhula variabilis Riess	DD
Tyromyces chioneus (Fr.) P.Karst.	LC
Wynnella silvicola Nannf.	LC
Xanthoporia radiata (Sowerby) Ţura, Zmitr., Wasser, Raats & Nevo	LC
Xerocomus longistipitatus K.Das, A.Parihar, D.Chakr. & Baghela	LC
